# Molecular basis of high-torque transmission of the *Vibrio* polar flagellar motor

**DOI:** 10.1093/procel/pwag025

**Published:** 2026-03-23

**Authors:** Ling Zhang, Jiaxing Tan, Xuemin Duan, Xiaofei Wang, Ting Wang, Keke Yuan, Michio Homma, Seiji Kojima, Yan Zhou, Yongqun Zhu

**Affiliations:** School of Public Health, Life Sciences Institute, and Department of Infectious Diseases of the Second Affiliated Hospital, School of Medicine, Zhejiang University, Hangzhou 310058, China; The MOE Key Laboratory of Biosystems Homeostasis & Protection, and Zhejiang Provincial Key Laboratory of Cancer Molecular Cell Biology, Life Sciences Institute, Zhejiang University, Hangzhou 310058, China; School of Public Health, Life Sciences Institute, and Department of Infectious Diseases of the Second Affiliated Hospital, School of Medicine, Zhejiang University, Hangzhou 310058, China; The MOE Key Laboratory of Biosystems Homeostasis & Protection, and Zhejiang Provincial Key Laboratory of Cancer Molecular Cell Biology, Life Sciences Institute, Zhejiang University, Hangzhou 310058, China; School of Public Health, Life Sciences Institute, and Department of Infectious Diseases of the Second Affiliated Hospital, School of Medicine, Zhejiang University, Hangzhou 310058, China; The MOE Key Laboratory of Biosystems Homeostasis & Protection, and Zhejiang Provincial Key Laboratory of Cancer Molecular Cell Biology, Life Sciences Institute, Zhejiang University, Hangzhou 310058, China; State Key Laboratory for Vegetation Structure, Function and Construction, Institute of Microbiology, College of Life Sciences, Zhejiang University, Hangzhou 310058, China; School of Public Health, Life Sciences Institute, and Department of Infectious Diseases of the Second Affiliated Hospital, School of Medicine, Zhejiang University, Hangzhou 310058, China; The MOE Key Laboratory of Biosystems Homeostasis & Protection, and Zhejiang Provincial Key Laboratory of Cancer Molecular Cell Biology, Life Sciences Institute, Zhejiang University, Hangzhou 310058, China; State Key Laboratory for Vegetation Structure, Function and Construction, Institute of Microbiology, College of Life Sciences, Zhejiang University, Hangzhou 310058, China; School of Public Health, Life Sciences Institute, and Department of Infectious Diseases of the Second Affiliated Hospital, School of Medicine, Zhejiang University, Hangzhou 310058, China; The MOE Key Laboratory of Biosystems Homeostasis & Protection, and Zhejiang Provincial Key Laboratory of Cancer Molecular Cell Biology, Life Sciences Institute, Zhejiang University, Hangzhou 310058, China; State Key Laboratory for Vegetation Structure, Function and Construction, Institute of Microbiology, College of Life Sciences, Zhejiang University, Hangzhou 310058, China; School of Public Health, Life Sciences Institute, and Department of Infectious Diseases of the Second Affiliated Hospital, School of Medicine, Zhejiang University, Hangzhou 310058, China; The MOE Key Laboratory of Biosystems Homeostasis & Protection, and Zhejiang Provincial Key Laboratory of Cancer Molecular Cell Biology, Life Sciences Institute, Zhejiang University, Hangzhou 310058, China; State Key Laboratory for Vegetation Structure, Function and Construction, Institute of Microbiology, College of Life Sciences, Zhejiang University, Hangzhou 310058, China; Department of Physics, Nagoya University, Nagoya 464-8601, Japan; Department of Biological Science, Graduate School of Science, Nagoya University, Nagoya 464-8601, Japan; Department of Biological Science, Graduate School of Science, Nagoya University, Nagoya 464-8601, Japan; School of Public Health, Life Sciences Institute, and Department of Infectious Diseases of the Second Affiliated Hospital, School of Medicine, Zhejiang University, Hangzhou 310058, China; State Key Laboratory for Vegetation Structure, Function and Construction, Institute of Microbiology, College of Life Sciences, Zhejiang University, Hangzhou 310058, China; School of Public Health, Life Sciences Institute, and Department of Infectious Diseases of the Second Affiliated Hospital, School of Medicine, Zhejiang University, Hangzhou 310058, China; The MOE Key Laboratory of Biosystems Homeostasis & Protection, and Zhejiang Provincial Key Laboratory of Cancer Molecular Cell Biology, Life Sciences Institute, Zhejiang University, Hangzhou 310058, China; Shanghai Institute for Advanced Study, Zhejiang University, Shanghai 310058, China; Cancer Center, Zhejiang University, Hangzhou 310058, China; New Cornerstone Science Laboratory, Shenzhen 518054, China

**Keywords:** polar flagella, flagellar motor, HT ring, hook, Leucine-rich repeat, *Vibrio*

## Abstract

The bacterial flagellar motor is a protein nanomachine that rotates the flagellum to facilitate bacterial motility. These motors exhibit structural diversity among species, enabling the transmission of varying torques to flagellar filaments to grant bacteria diverse swimming capabilities. Compared to peritrichous flagellar motors, polar flagellar motors are faster machines that transmit higher torque to drive high-speed motility in liquids and empower swimming in viscous environments. However, structural basis of high-torque transmission of the polar flagellar motors is still unclear. Here we present a cryo-electron microscopy structure of the polar flagellar motor in complex with the hook from *Vibrio alginolyticus*, comprising 295 subunits from 18 proteins. Compared to the peritrichous flagellar rod, this structure reveals an increased number of inter-subunit interactions in the rod of the polar flagellar motor. Nine phospholipid molecules insert into the interface between the export apparatus and the proximal rod. The LP ring contains more electrostatic charges on the inner surface and has fewer physical contacts with the rod, while the HT ring tightly binds to the outer surface of the LP ring. FlrP, a protein of previously unknown function, is identified as a new component of the polar flagellar motor, and extensively participates in the interactions of 15 FliF peptides of the MS ring with the rod. In contrast to that in the peritrichous flagellum, the hook in the polar flagellum has two different conformational states, L- and R-types. These findings provide unprecedented insights into structural adaptations of the bacterial polar flagellar motors for high-torque transmission.

## Introduction

Bacterial motility is required for environmental survival and the virulence of many bacteria species (Moens and Vanderleyden, 1996; Subramanian and Kearns, 2019). The flagellum is a complex protein machine mediating bacterial swimming and swarming motility (Armitage and Berry, 2020; Nakamura and Minamino, 2019). The structure of the bacterial flagellum contains three parts: the rotary motor, the hook and the filament (Berg, 2003; Carroll and Liu, 2020). The flagellar motor spans the bacterial membranes and is powered by ion-conducting stators, MotAB or PomAB (Santiveri et al., 2020). Serving as the assembly base of the flagellum, the motor usually consists of the L, P, MS, and C rings, the rod, and the export apparatus (Nakamura and Minamino, 2019). The hook is a universal joint connecting the motor to the helical filament, which functions as a propeller to drive the bacterial cell movement. Flagellar arrangements vary significantly, including monopolar, lophotrichous, amphitrichous, and peritrichous. The majority of bacteria on Earth are polarly flagellated, possessing one or more flagella at the cell poles (Homma et al., 2022; Lloyd and Klose, 2023; Thormann et al., 2022), such as *Vibrio alginolyticus*, *Pseudomonas aeruginosa*, *Campylobacter jejuni*, and *Helicobacter pylori* (Cohen et al., 2024; Leifson et al., 1964; Zhu et al., 2019). In contrast to the peritrichous flagellar motors, which transmit low torque (about 1,300 pN nm in *Escherichia coli* and *Salmonella* Typhimurium) generated by the H^+^-driven MotAB stators (Guo and Liu, 2022; Magariyama et al., 1994; Nakamura, 2020), the polar flagellar motors utilize the Na^+^-driven PomAB stators and transmit substantially higher torque (about 3,800 pN nm in *V*. *alginolyticus*), enabling the rotation of the polar flagellum at speeds up to 1,700 revolutions per second (Magariyama et al., 1994) and facilitating bacterial swimming in viscous environments.

We previously solved the cryo-electron microscopy (cryo-EM) structure of the peritrichous flagellar motor of *S*. Typhimurium, which consists of the C26-symmetric LP ring in the outer membrane, the MS ring in the inner membrane, the rod, the export apparatus, the cytoplasmic C ring, and the stator units that generate torque by transducing protons or sodium ions from the extracellular side to the cytoplasm (Tan et al., 2021, 2024). To achieve high-torque transmission, the polar flagellar motor has evolved additional components, resulting in a more complex architecture than its peritrichous counterpart (Beeby et al., 2016; Homma et al., 2022; Lloyd and Klose, 2023). For instance, in *Vibrio* species, additional H and T rings surround the LP ring to form an LPHT ring in the outer membrane (Takekawa et al., 2024; Zhu et al., 2017, 2020). Despite significant progress in cryo-electron tomography imaging and other structural efforts, the detailed structural adaptation and molecular basis of high-torque transmission of the bacterial polar flagellar motors are still unclear.

In addition, the polar flagellum of *V*. *alginolyticus* drives a unique “forward-reverse-flick” motility pattern of the bacteria for cell reorientation, which differs significantly from the typical “run-and-tumble” pattern of the peritrichously flagellated bacteria (Kinosita and Sowa, 2023; Thormann et al., 2022; Wadhwa and Berg, 2022). The flicking behavior of the bacteria relies on a buckling instability process of the hook (Son et al., 2013; Xie et al., 2011). Generally, the polar flagellar hook adopts a straight conformation to transmit torque from the motor to the filament, pushing the cell body forward during counterclockwise (CCW) rotation or pulling it backward during clockwise (CW) rotation (Homma et al., 2022; Thormann et al., 2022). However, in the flicking behavior, the straight hook bends under the thrust forces from the flagellar filament upon the change of the flagellar rotational direction from CW to CCW (Jabbarzadeh and Fu, 2020; Shum and Gaffney, 2012; Son et al., 2013). During this bending process, the FlgE subunits at the innermost curvature of the hook undergo potential structural collapses. It remains unknown how the hook maintains the structural flexibility required for bending while overcoming these potential collapses. In this study, we determined a cryo-EM structure of the polar flagellar motor-hook complex from *V*. *alginolyticus*, providing unprecedented insights into the structural evolution and molecular basis for high-torque transmission of the polar flagellar motor.

## Results

### Overall structure of the polar flagellar motor-hook complex

In *V. alginolyticus*, FlgL is an essential adapter protein responsible for connecting the hook to the filament of the polar flagellum (Lloyd and Klose, 2023; Uesaka et al., 2024). To isolate polar flagellar motor particles without interference from filaments or lateral flagella, we generated a Δ*flgL* mutant strain of *V. alginolyticus* KK148 that is incapable of produce the lateral flagella but successfully assemble multiple polar flagella at the cell pole without filaments (Kusumoto et al., 2006, 2008; Uesaka et al., 2024). The particles of the polar flagellar motor-hook complex of *V. alginolyticus* were successfully purified from the Δ*flgL* mutant strain (Tan et al., 2021) and contained the LPHT ring, the MS ring, the rod, the export apparatus, and the hook (Figs. 1A and S1A). To determine the structural symmetry of the LPHT ring, we further disassembled the purified motor–hook complexes by reducing the salt concentration in the purification buffer. The resulting LPHT ring particles remained highly stable despite motor disassembly, allowing for high-resolution cryo-EM data analysis and symmetry determination (Figs. S1A, S1B and S1D).

**Figure 1.**
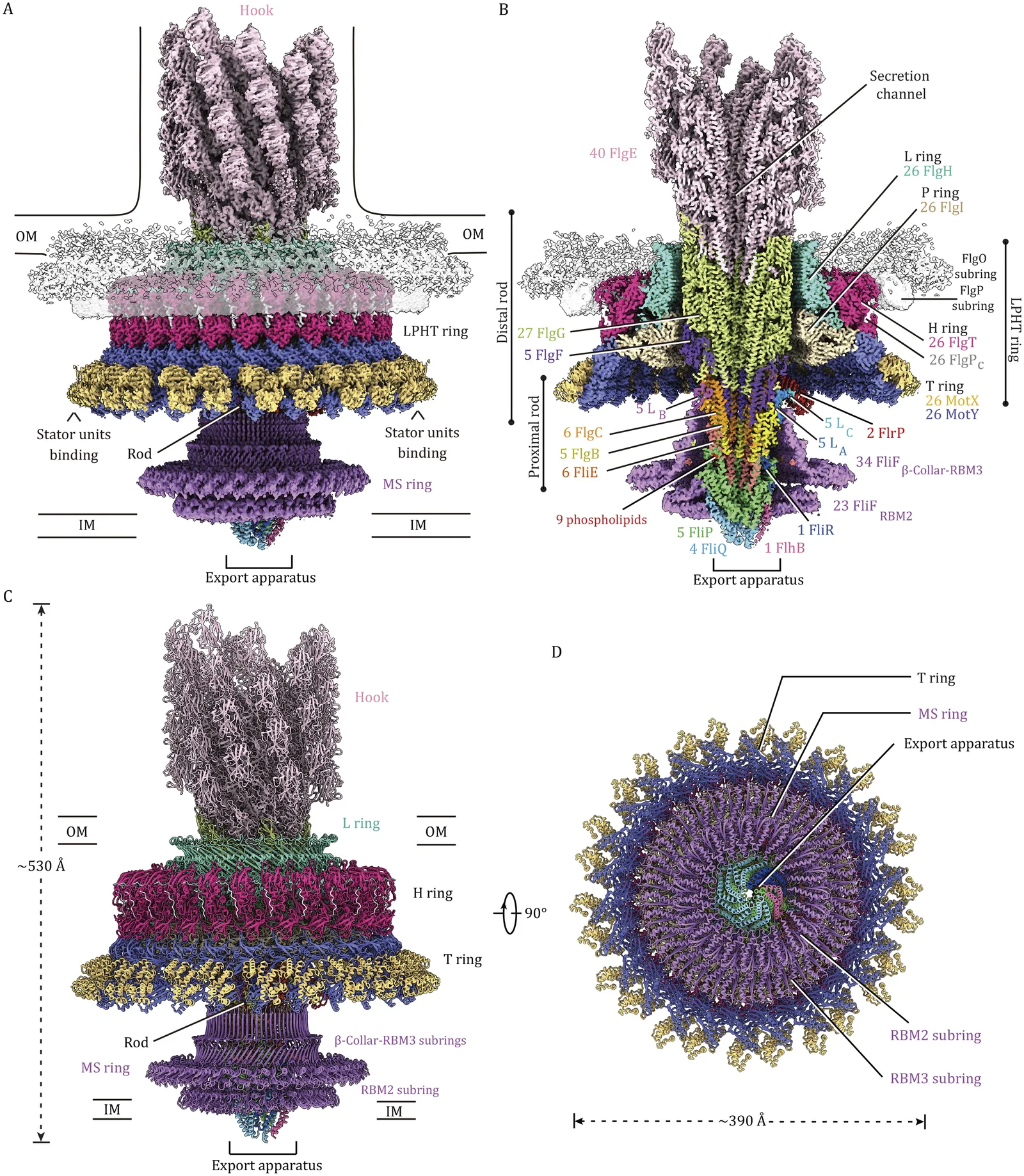
**Overall structure of the polar flagellar motor-hook complex of *V. alginolyticus***. (A and B) The side (A) and cross-section (B) views of the merged density map of the polar flagellar motor-hook complex after local refinements. The map was obtained by fitting the high-resolution density maps of six locally refined regions, including the LPHT ring, the hook (L-type), the distal rod, the proximal rod with the export apparatus, the β-collar-RBM3 subrings (C34 symmetry) and the RBM2 subring (C1 symmetry) of the MS ring into the global density map of the polar flagellar motor-hook complex reconstructed with C100 symmetry. Semi-transparent illustration of the densities of the FlgP and FlgO subrings is applied to show the overall structure of the LPHT ring. The subunit numbers of the components in the complex are labeled as indicated (B). In the MS ring, all β-collar and RBM3 domains of the 34 FliF subunits were found in the β-collar-RBM3 subring, while only 23 RBM2 domains of FliF were identified in the RBM2 subring. All three domains of FliF are colored in purple (B). (C and D) The side (C) and bottom views (D) of the final structural model of the polar flagellar motor-hook complex. OM, outer membrane; PG, peptidoglycan; IM, inner membrane.

A clear density map of the entire motor-hook complex was successfully obtained and allowed us to assemble our locally refined density maps of different parts of the complex into the volume for overall structural modeling (Fig. S1C). Two-dimensional (2D) classification of the end-on views of the LPHT ring particles revealed 26-fold symmetry of the LPHT ring (Fig. S1D). Further three-dimensional (3D) classification of the LPHT ring particles revealed an unexpected 13-fold symmetry in its lower region, indicating a mismatch between the 26- and 13-fold symmetries within the LPHT ring (Fig. S1C). Reconstruction with C13 symmetry yielded a density map of the LPHT ring with a resolution of ∼2.95 Å, facilitating *de novo* model building of each subunit (Figs. S1C and S2). At the top region of the H ring, there are two layers of ring-like, smeared densities. These regions could not be structurally modeled and are possibly the FlgO subring and the lipid-bound region of the FlgP subring, respectively, according to the previous cryo-electron tomography (cryo-ET) analyses (Zhu et al., 2017, 2020) (Fig. 1A and 1B).

The hook in the complex particles was in two distinct conformational states (Fig. S1C), hereafter referred to as the L- and R-type, respectively. These states are characterized by the left- and right-handed helical arrangements of the D2 domains of the FlgE subunits at the outermost layer. The density maps of the L- and R-hooks were reconstructed to ∼3.2 Å and ∼3.4 Å, respectively (Figs. S1C and S2). Local refinement of the MS ring without imposed symmetry produced a ∼3.6 Å density map, revealing 34-fold and 23-fold symmetries in the upper β-collar-RBM3 and lower RBM2 subrings, respectively (Figs. S1C and S2). The density map of the β-collar-RBM3 subring of the MS ring was finally reconstituted to ∼2.9 Å with C34 symmetry (Figs. S1C and S2). Focused refinement also produced high-resolution density maps for other regions, including the axis region (comprising the rod, the export apparatus, and a part of the hook), the proximal rod region with the export apparatus, and a middle part of the rod, to the resolutions of ∼3.0 Å, ∼3.0 Å, and ∼3.5 Å, respectively (Figs. S1C and S2).

The final model of the polar flagellar motor-hook complex contains 295 subunits from 18 proteins, including 26 subunits of each component of FlgH, FlgI, FlgT, MotX, MotY, and FlgP in the LPHT ring; 34 FliF subunits in the MS ring; 1 FlhB, 4 FliQ, 5 FliP and 1 FliR subunits in the export apparatus; 6 FliE, 5 FlgB, and 6 FlgC subunits in the proximal rod; 5 FlgF and 27 FlgG subunits in the distal rod; 40 FlgE subunits in the part of the hook; and 5 subunits of a previously function-unknown leucine-rich repeat (LRR) proteins (hereafter named FlrP), which are bound onto the middle region of the rod (Figs. 1A–C). In the complex, the LPHT ring encircles the distal rod, while the MS ring encloses the export apparatus and the proximal rod (Figs. 1A and 1B). The complete structural model of the polar flagellar motor-hook complex has a molecular weight of ∼10 MDa, with an outermost diameter of ∼390 Å at the T ring and a total height of ∼530 Å. The mass and dimensions of this polar flagellar motor significantly exceed those of the *Salmonella* peritrichous flagellar motor (Figs. 1C and 1D).

### The HT ring

In the motor-hook complex, the LP ring consists of 26 FlgH and 26 FlgI subunits with an inner diameter of ∼130 Å to embrace the distal rod (Fig. 2A). Each FlgH subunit interacts with the three preceding and three succeeding FlgH protomers, while each FlgI subunit interacts with the two preceding and two succeeding FlgI protomers (Figs. S3B and 3C). FlgH harbors a distinctive N-terminal loop (L_N_ loop) that is significantly longer than its counterpart in peritrichous flagella and adopts a more compact conformation in its middle and C-terminal regions (Figs. 2A, 2B, and S3A–D). This results in a “1-versus-3” interaction between FlgH and FlgI subunits at the L–P interface, which is relatively looser than the “1-versus-4” arrangement observed in peritrichous motors (Figs. S3G and S3H). The N-terminus of the L_N_ loop extends outward to interact with the FlgT and FlgP subrings of the H ring (Figs. 2B, 2E, and S5C).

**Figure 2.**
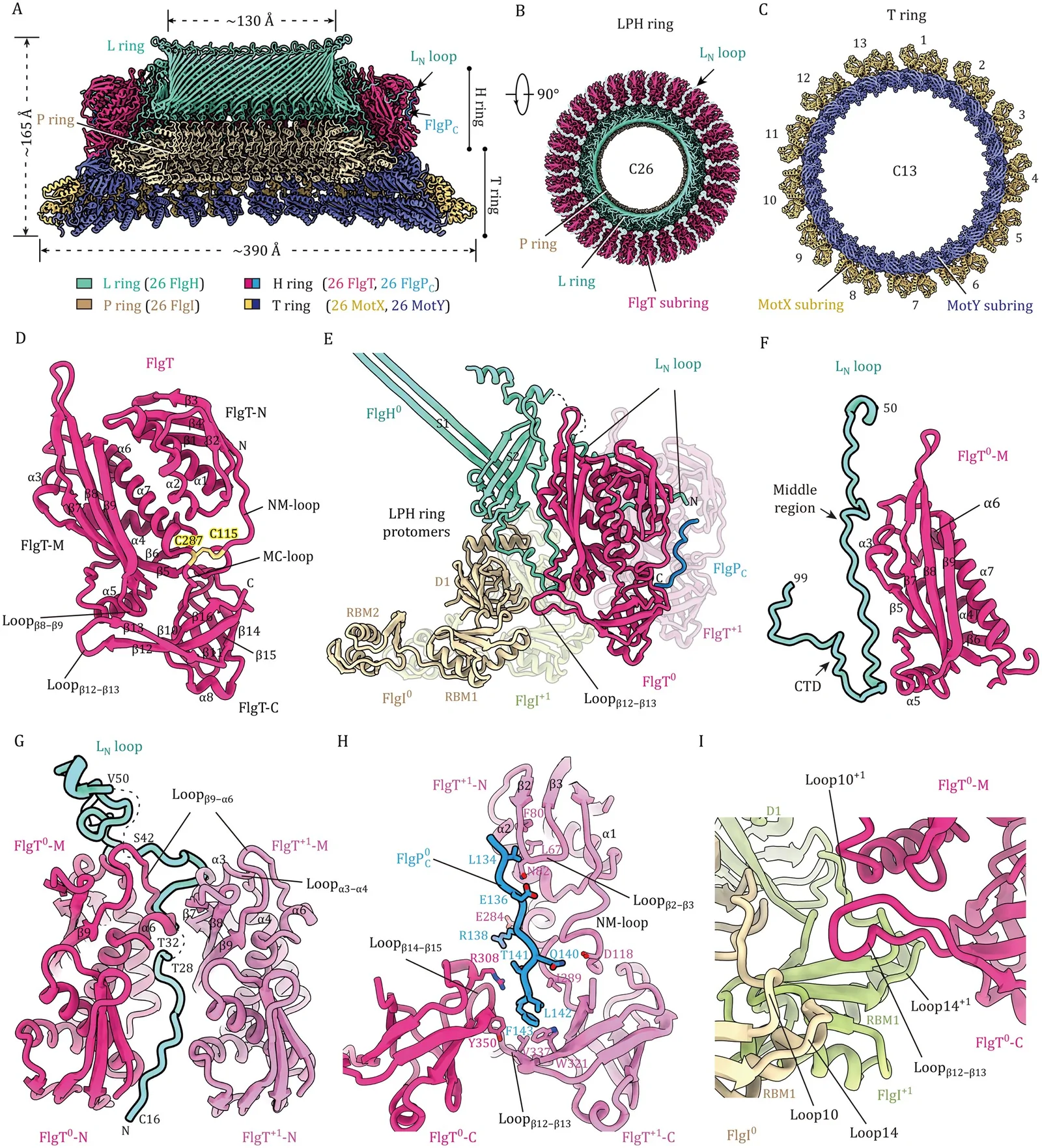
**The structure and inter-subunit interactions of the LPHT ring**. (A) Cross-section view of the structure of the LPHT ring in the polar motor-hook complex. Each ring and its components are colored as indicated. The L_N_ loop of a FlgH subunit is colored in cyan and highlighted with a black arrow. (B) Top view of the structure and C26 symmetry of the LPH ring. The FlgT subring of the H ring is highlighted with a black line. (C) Top view of the structure and C13 symmetry of the T ring. The MotX and MotY subunits are colored in yellow and blue, respectively. (D) The FlgT structure in the LPHT ring. The three domains (FlgT-N, FlgT-M, FlgT-C) of FlgT are labeled as indicated. The disulfide bond between C115 and C287 in FlgT is highlighted in yellow. (E) Interactions of FlgT with the subunits of FlgH, FlgI, and FlgP_C_ in the LPHT ring. The subunits in the different protomers are labeled with numbers. FlgT, FlgH, FlgI, and FlgP_C_ are colored in red, cyan, yellow, and deep blue, respectively. (F) Interactions of the middle region (residues 50–99) of the L_N_ loop of FlgH with the FlgT subunits in the LPHT ring. (G) Interactions of N-terminal regions (residues 16–49) of the L_N_ loop of FlgH with the FlgT subunits in the LPHT ring. The flexible regions (residues 29–31 and residues 43–48) of the L_N_ loop are indicated with dashed lines. (H) Detailed interactions of FlgP_C_ with the FlgT subunits. The FlgP_C_ binds into an open groove in the FlgT subring. (I) Interactions between the FlgT subring and the P ring. The loop_β12–β13_ of FlgT-C interacts with the loops 10 and 14 of FlgI.

The H and T rings surround the LP ring and consist of 26 FlgT and 26 FlgP, and 26 MotX and 26 MotY subunits, respectively (Fig. 2A–C). In the H ring, FlgT consists of three domains (FlgT-N, FlgT-M and FlgT-C) and adopts a closed conformation, contrasting with the open conformation seen in its *apo* crystal structure (Terashima et al., 2013) (Figs. 2D, S5A, and S5B). Unlike the C26 symmetry of the LP and H rings, the T ring adopts C13 symmetry (Zhu et al., 2020) (Figs. 2B, 2C and S4A). Each protomer of the T ring contains two MotX-Y heterodimers (MotX_1_-Y_1_ and MotX_2_-Y_2_) (Figs. S4B–E and S5D), in which the C-terminal regions of MotX that are essential for the interactions of the two MotX-Y dimers in the protomer (Fig. S4D) are in two distinct conformations. The C-terminal region of MotX_2_ extends from its main body to insert into a hydrophobic cavity in MotX_1_-C (Figs. S4F and S4G), whereas the C-terminal region of MotX_1_ folds back into a cleft between α4 of MotY_1_-C and α9 of MotX_1_ in the MotX_1_-Y_1_ dimer (Fig. S4E). In the T ring, the N-terminal domains of MotY (MotY-N) interact with each other in a head-to-tail fashion (Fig. S4B and S4H). MotX bridges the two subrings of the T ring by interacting with the upper two MotY-N domains (Fig. S4J).

The HT and LP rings generate extensive interactions. The L_N_ loops of FlgH clamp the FlgT-M domains in the H ring (Figs. 2A, 2B, 2E, and 2G). The middle region of L_N_ binds to α3, α5, the C-terminus of β5, and loop_α3–α4_, loop_β6–α5_, and loop_β9–α6_ of FlgT-M (Fig. 2F). The T32-S52 fragment at the N-terminal region of L_N_ extends outwards and inserts into a groove formed by α6 and loop_β9–α6_ of FlgT-M with the loop_α3–α4_ and β7 of FlgT^+1^-M (Fig. 2G). The proximal region (residues C16-T28) of L_N_ binds into the deep cleft between the adjacent FlgT-NM domains (Fig. 2G). The invisible lipid-modified N-terminus of the L_N_ loop likely functions as an anchor with the FlgP subring to anchor the LPHT ring to the outer membrane (Fig. S5C). The C-terminal tail (residues Q133-F143) of each FlgP subunit obliquely binds into an open groove in the FlgT subring (Fig. 2H). Additionally, the loop_β12–β13_ of FlgT-C interacts with the loops 10 and 14 of FlgI at the H-LP ring interface (Fig. 2I). MotY-N inserts into the cleft between the FlgI-RBM1 and FlgT-C domains, further stabilizing the P ring (Fig. S4I).

### Phospholipids and inter-subunit interactions in the polar rod

In contrast to the 24 FlgG subunits found in the *Salmonella* peritrichous rod, the distal rod of the *V. alginolyticus* polar motor contains 27 FlgG subunits (Fig. 3A). The polar rod is located on the export apparatus, which is bound by two N-terminal transmembrane helices of FlhB from the bottom (Figs. 3A, 3B, and S6A). The export apparatus adopts an expanded configuration to accommodate the proximal rod subunits (Figs. 3B and S6B). Notably, nine phospholipid molecules, likely phosphatidylglycerols and phosphatidylethanolamines, are found at the interface of the export apparatus and the proximal rod (Figs. 3B–D). These phospholipids insert into the clefts formed by FliE^2^-FlgB^2^-FliP^2^, FliE^3^-FlgB^3^-FliP^3^, FliE^4^-FlgB^4^-FliP^4^, FliE^5^-FlgB^5^-FliP^5^, and FliR-FliE^6^-FliE^1^-FlgC^1^, respectively (Figs. 3B, 3D, and S6M) and act as molecular glue to strengthen the interactions between the export apparatus and the proximal rod.

**Figure 3.**
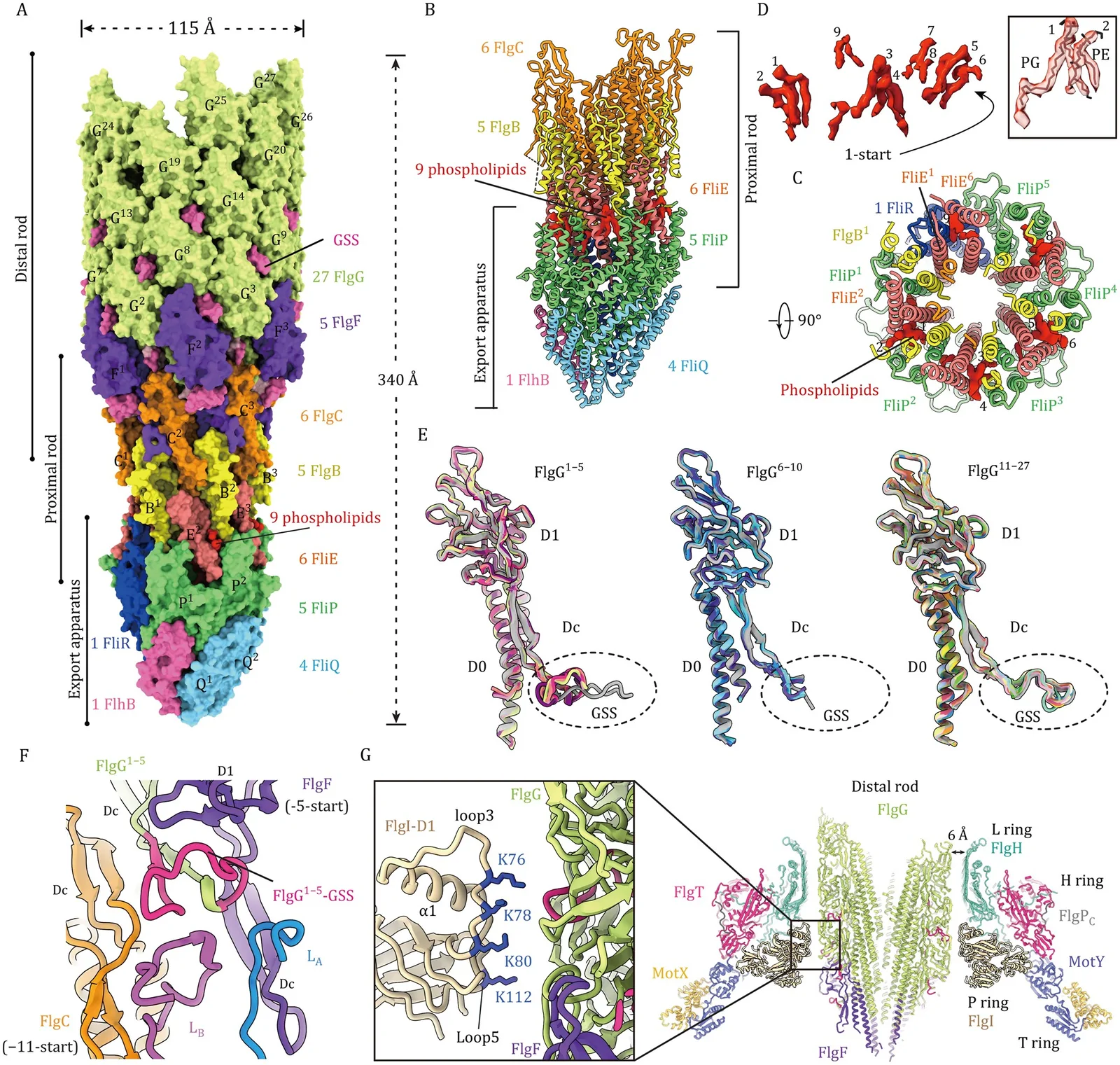
**The structure of the rod**. (A) Overall structure of the rod with the export apparatus in the polar flagellar motor-hook complex. The rod and the export apparatus are represented in surface. The subunit numbers of the components are labeled as indicated. The GSS regions of the FlgG subunits are highlighted in red. (B and C) Side (B) and top views (C) of the binding of nine phospholipids into the grooves at the interface of the proximal rod and the export apparatus. (D) Cryo-EM density maps of nine phospholipid molecules. The densities of the nine phospholipids are colored in red (B, C, and D). Close-up views of the first and second phospholipids are shown at right and modeled as PG and PE molecules, respectively. (E) Structural comparison of the FlgG subunits in the rod. The FliG subunits are classified into three classes, FliG^1–5^, FliG^6–10^, and FliG^11–27^, based on the conformations of their GSS regions. (F) Interactions of the GSS regions of the FlgG^1–5^ subunits with the adjacent subunits in the rod along the −11- and −5-start directions. The GSS region is in a compressed conformation. (G) Interactions of the P ring with the rod. The lysine residues of the D1 domain of FlgI, which are involved in the interactions with the rod, are shown as sticks and colored in blue. Each component of the LPHT ring is listed as indicated. The distance of the L ring to the rod is about 6 Å, which is highlighted with a dashed line.

The subunits of the polar rod form more inter-subunit interactions than those in the peritrichous rod. Each polar rod protein harbors unique structural features (Figs. S6D–G). FliE contains an elongated α2 helix for interaction with the export apparatus (Fig. S6D). The N-termini of the α_N_ helices of the FlgB subunits twist to interact with the upper regions of the FliP subunits, thereby enhancing interactions between the export apparatus and the proximal rod (Fig. S6E). FlgC has a unique lip structure to interact with the FliF peptide loops and the newly identified LRR proteins, FlrPs (discussed below) (Fig. S6F). Unlike *Salmonella* FlgF, the polar FlgF lacks an α3 helix (Fig. S6G). The FliG subunits in the polar rod can be classified to three groups: FliG^1–5^, FliG^6–10^, and FliG^11–27^, based on the conformations of their FlgG-specific sequence (GSS) regions (Fig. 3E). The GSS regions of FlgG^1–5^ adopt a compressed conformation and extensively interact with the FlgC^1–5^-Dc and FlgF^1–5^-Dc-D1 domains, as well as with the FliF peptide loops (L_A_ and L_B_) along the −11- and −5-start directions (Figs. 3E and 3F). While the GSS regions of FlgG^6–10^ appear unfolded, the FlgG^11–27^ GSS region interacts extensively with the neighboring FlgG and FlgF subunits in the −16, −10, and −5 directions (Fig. S6H). FlgG^11^ also interacts with FlgC^6^ at the −5-start position (Fig. S6H).

### The polar LP ring-rod interface

At the LPHT ring-rod interface, physical contact is limited to the P ring, which interacts with the D1 domains of FlgF^1–5^, the D1 domains of FlgG^1–10^, and the GSS regions of FlgG^14–18^ via four lysine residues (K76, K78, and K80 in loop3 and K112 in loop5) within the FlgI-D1 domains (Fig. 3G). This contrasts with the *Salmonella* peritrichous motor, where the P ring utilizes two lysine residues and five polar residues (glutamine and asparagine) in FlgI-D1to form a hydrogen-bond ring with the rod (Tan et al., 2021). Notably, the buried surface area between the P ring and the rod in the *Vibrio* polar flagellar motor is 1,781.2 Å^2^, significantly <4,913.6 Å^2^ observed in the *Salmonella* peritrichous flagellar motor, indicating far fewer physical contacts between the rod and the LPHT ring (Figs. 3G and S3J). Furthermore, the distal rod surface carries a greater abundance of negatively charged residues with nine aspartates and ten glutamates on each D1 domain of the FlgG subunits, compared to six aspartates and five glutamates on the peritrichous counterpart (Fig. S6I–L). The L ring in the polar flagellar motor also carries more negatively charged residues with six aspartates and two glutamates on the rod-facing surface of each FlgH subunit, compared to just two aspartates and one glutamate on each FlgH surface of the peritrichous motor (Figs. S6I–L). These observations suggest that the LP ring utilizes enhanced electrostatic interactions and less physical contacts to stabilize the rod during its rotation at higher speeds.

### The MS ring–rod interactions in the polar motor

Similar to the peritrichous motor, the MS ring in the polar flagellar motor exhibits a symmetry mismatch between the upper C34-symmetric RBM3-β-collar subring and the lower C23-symmetric RBM2 subring (Takekawa et al., 2024) (Figs. S7A and S7B). The MS ring has an outermost diameter of 230 Å at the RBM3 subrings (Fig. 4A). The lower RBM2 subring binds to the export apparatus via two long loops (loop 1 and loop 2) of each RBM2 domain (Fig. S7C). The RBM3-β-collar subring encompasses both the upper region of the export apparatus and the lower region of the proximal rod (Fig. 4A). The N-terminal α1 helices of the six FliE subunits and the Dc loops of the five FlgB subunits extend outward from the proximal rod to bind the inner surface of the β-collar subring of the MS ring (Fig. 4A).

**Figure 4.**
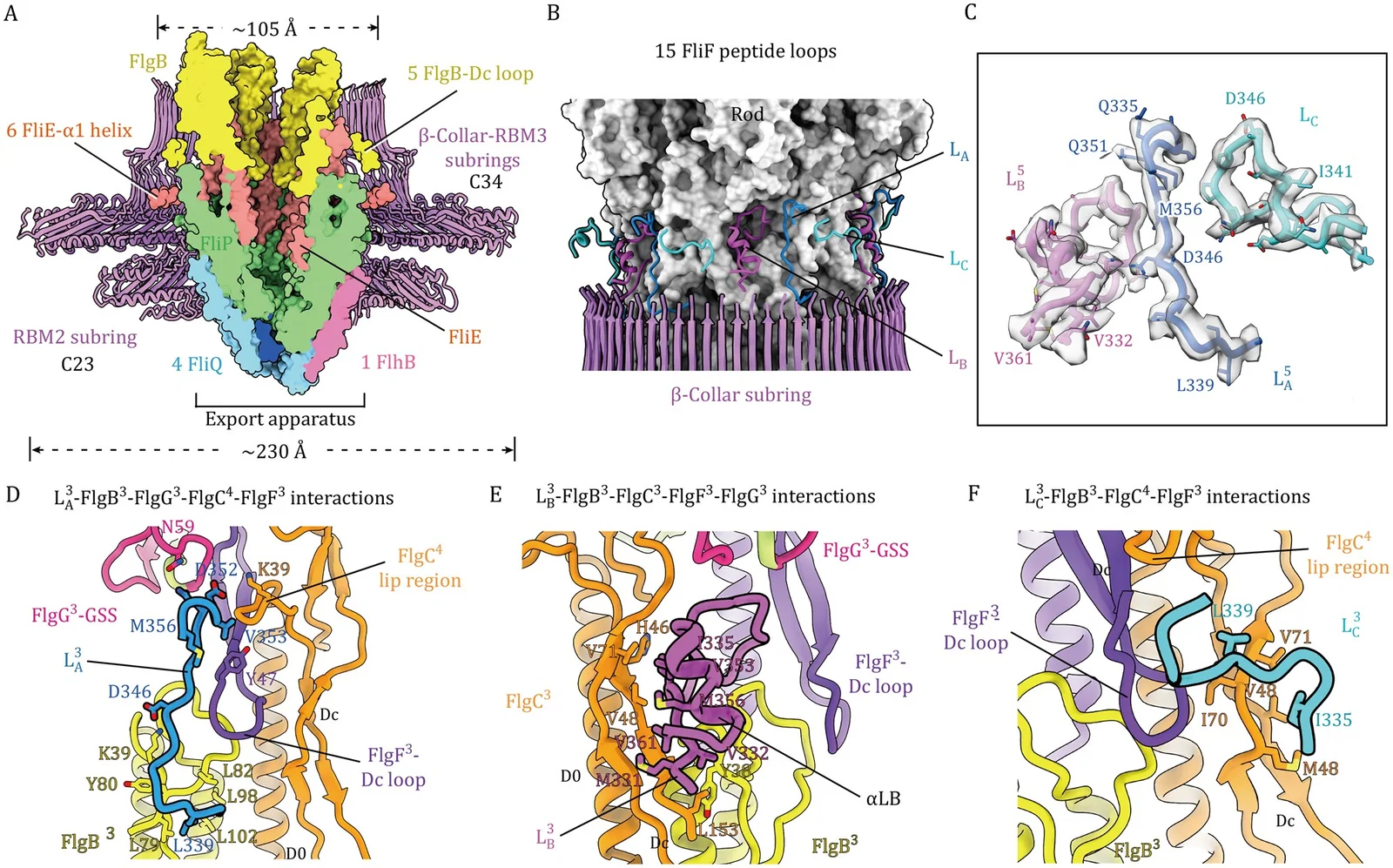
**The MS ring-rod interactions**. (A) Structure of the MS ring and its interactions with the proximal rod and the export apparatus. The MS ring is represented in a cartoon. The proximal rod and the export apparatus are shown in surface. The N-terminal α1 helices of the six FliE subunits and the Dc loops of the five FlgB subunits bind to the inner surface of the β-collar subring of the MS ring. (B) Interaction of the 15 FliF peptide loops with the rod. The rod is represented in a surface. The β-collar subring of the MS ring is shown in a cartoon. The L_A_, L_B_, and L_C_ loops of FliF are colored in blue, purple, and cyan, respectively. (C) Representative cryo-EM densities of L_A_, L_B_, and L_C_. The structural models of the $L_{A}^{5}$ loop, $L_{B}^{5}$ loop, and $L_{C}^{5}$ loop are illustrated. (D–F) Detailed interactions of the L_A_ (D), L_B_ (E), and L_C_ loops (F) with the subunits of the rod. The $L_{A}^{3}$, $L_{B}^{3}$, and $L_{C}^{3}$ loops with the rod are colored in blue, purple, and cyan, respectively. The side chains of the interacting residues are shown as sticks. The interacting subunits of the rod are shown in a cartoon and labeled as indicated.

Locally refined maps of the proximal and middle rod revealed that 15 FliF peptide loops extending upward from the β-collar subring of the MS ring to bind the rod for torque transmission (Fig. 4B). This is a significant increase from the 11 extended FliF peptide loops observed in the peritrichous flagellar motor(Tan et al., 2021, 2024) (Fig. S7E). These 15 loops are grouped into five sets, each comprising an L_A_, L_B,_ and L_C_ loop and packed helically on the rod’s surface (Figs. 4B, 4C, and S7D). The L_A_ loop is bound into the long clefts formed by the upper region of FlgB^1-5^-Dc domain with the FlgF^1–5^-Dc, FlgC^2–6^-lip, and FlgG^1–5^-GSS regions at the 11-, 6- and 16-start positions (Fig. 4D). The L_B_ loop, which harbors a unique α_LB_ helix, inserts to an open pocket generated by the upper region of FlgB^1–5^-Dc, the D0 and D1 domains of FlgC^1–5^ and FlgF^1–5^, and the FlgG^1–5^-GSS at the −11-, 5-, 11-, and 16-start positions, respectively (Fig. 4E). The L_C_ loop interacts with the concave surface on the lower region of FlgF^1–5^-Dc and the middle region of FlgC^1–5^-Dc (Fig. 4F). The N-terminus of L_C_ interacts with the α_LB_ helix of $L_{B}^{+1}$ of the succeeding set, while the C-terminus of L_C_ contacts with the L_A_ loop and the lip region of FlgC^1–5^ (Fig. 4B), thereby reinforcing the inter-loop interactions of the 15 FliF peptide loops on the surface of the rod.

### A new component in the polar motor

In addition to the 15 FliF loops, the 3.5 Å-resolution local density map of the proximal rod revealed five β-solenoid-like structures encircling the rod (Fig. S1C). Mass spectrometry identified a previously uncharacterized protein of 21.5 kDa (protein ID: BCB49735.1, hereafter named FlrP) within the purified particles of the polar flagellar motor-hook complex (Fig. S8A). FlrP contains an N-terminal Sec signal peptide (residues M1-A19) and is a typical leucine-rich repeat (LRR) protein. Western blotting using a monoclonal antibody specific to FlrP further confirmed its presence in the polar flagellar motor (Fig. S8B). Among the five FlrP subunits, FlrP^1^ and FlrP^5^ exhibited high-quality densities, allowing for *de novo* structural modeling of all residues (Figs. 5A, 5B, and S8C). The FlrP structure consists of a β-solenoid LRR repeat core with an N-terminal head (residues Y20-Y34) and a C-terminal tail (residues G195-P199) (Figs. 5B and S8D). Structural analysis revealed that the FlrP subunits not only bind to the rod, but also interact with the L_B_ and L_C_ loops of the FliF subunits to strengthen the MS ring-rod interactions (Figs. 5D–G). The head regions of FlrP^1^ and FlrP^5^ bind into the clefts between the lip and cap regions of FlgC^2^ and FlgC^6^, and interact with the GSS regions of FlgG^2^ and FlgG^6^ and the D1 domains of FlgF^2^ and FlgG^1^, respectively (Figs. 5D–G). FlrP^1^ and FlrP^5^ also interact with the $L_{C}^{1}$ and $L_{C}^{5}$ loops, respectively, through coils 4, 5, and 6. FlrP^1^ interacts with α_LB_ of the $L_{B}^{2}$ loop through coil 1 and 2 (Figs. 5D–G).

**Figure 5.**
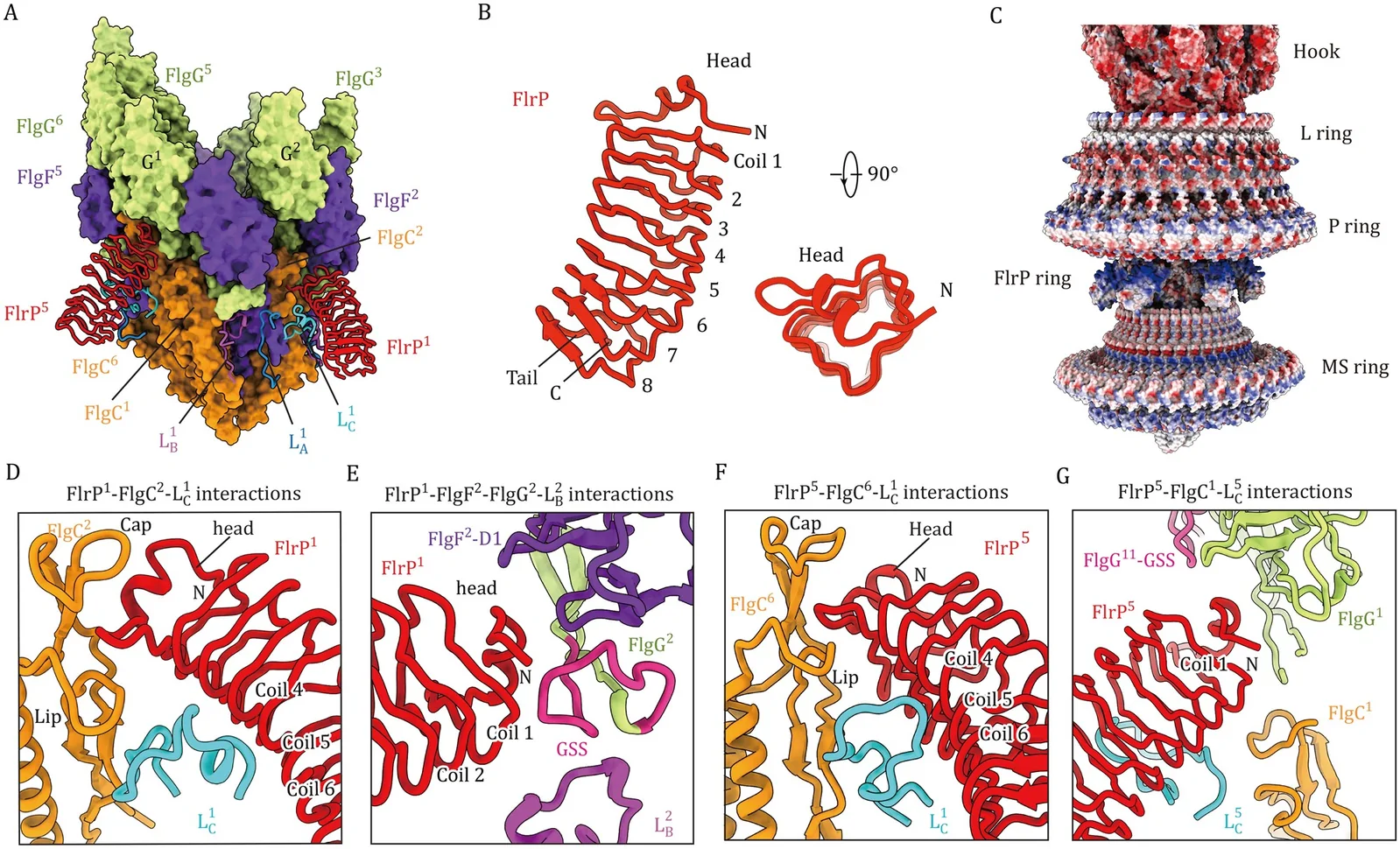
**The FlrP protein and its structure**. (A) Interactions of the FlrP subunits with the rod and the FliF peptide loops. The representative interactions of the FlrP^1^ and FlrP^5^ subunits with the rod and the FliF peptide loops are illustrated. The FlrP subunits and the L_A_, L_B,_ and L_C_ loops of FliF are represented in a cartoon. The components of the rod are shown in surface. (B) Structure of FlrP. The FlrP structure consists of a β-solenoid LRR repeat core with an N-terminal head (residues Y20–Y34) and a C-terminal tail (residues G195–P199). (C) Electrostatic potential distribution of the hook, the LP ring, the FlrP subunits, and the MS ring in the polar flagellar motor. The surface is colored by relative electrostatic potential, with red indicating negatively charged and blue positively charged. The FlrP subunits form a ring around the rod. (D–G) Representative interactions of the FlrP subunits with the rod and the FliF peptide loops. The interactions of the FlrP^1^ (D and E) and FlrP^5^ (F and G) subunits with the rod and the L_B_ and L_C_ loops of FliF are illustrated, respectively. The interacting subunits of the components of the rod are labeled as indicated.

The five FlrP subunits are helically packed on the surface of the rod, following the same helical parameters as the rod between the LPHT ring and the MS ring. They form a spiral ring with an outer diameter of ∼160 Å, which is much larger than the inner diameters of the RBM2 subring (∼145 Å) and the β-collar subring of the MS ring (∼105 Å) (Figs. S8E–G). Interestingly, the upper surface of each FlrP subunit is highly positively charged with the residues K54, K66, K69, R88, K92, K94, R109, K112, K130, R132, and K134, and produce electrostatic repulsion against the inner lower surface of the P ring that is also positively charged (Figs. 5C and S8H–J), suggesting that the FlrP subunits not only stabilize the FliF loop-rod interactions, but also likely act as charged spacers to maintain the positions of the LPHT and MS rings during the high-speed rotation of the polar motor (Fig. 5C). Functionally, the gene deletion of *flrP* significantly reduced the motility of *V. alginolyticus* (Figs. S9A and S9E). FlrP is conserved across 13 species of the *Vibrionaceae* family, including *Vibrio*, *Photobacterium*, *Aliivibrio*, *Salinivibrio*, *Grimontia*, and *Enterovibrio* (Fig. S9C). Its homologues have similar structures and harbor highly conserved rod- and L_B_/L_C_-interacting residues (Fig. S9D). Gene deletion of the FlrP homologue in *V. parahaemolyticus* also reduced the motility of the bacteria (Figs. S9B and S9F). In contrast to most of the flagellar genes that are generally located on chromosome I (McCarter, 2004; Okada et al., 2005), the *flrP* gene is located on chromosome II of *V. alginolyticus* and *Vibrio* species, indicating a conserved inter-chromosomal regulatory mechanism in the polar flagellar system.

### Two conformational states of the hook

In contrast to the curved hook in the peritrichous flagellum (Wadhwa and Berg, 2022), cryo-EM imaging analysis revealed the presence of two types of the straight hook, L- and R-type, in the particles of the polar motor-hook complex (Figs. 6A, 6B, S1A, and S1C). Approximately 63.2% of the particles harbor the L-type hook, while about 36.8% have the R-type hook (Fig. S1C). In both types of the hook, the FlgE subunits of *V. alginolyticus* assemble helically along the 1-start helical direction in a right-handed manner, and generate a three-layered tubular structure through the D0, Dc-D1, and D2 domains, respectively (Figs. 6A and 6B). The structures of the inner and middle tubes of the L-type hook are the same as those in the R-type hook (Figs. 6A and 6B). However, relative to the L-type hook, the outer tube of the R-type hook is in compressed conformation (Figs. 6A, 6B, and S10G). In the R-type hook, the D2 domains of the FlgE subunits (R-FlgE) pack via a head-to-tail interaction manner along the 6-start direction in the outer tube, which resembles the assembly of the D2 domains of the *Salmonella* FlgE subunits in the peritrichous curved hook (Figs. 6A, 6C, and S10A), whereas in the L-type hook, the D2 domains of the FlgE subunits (L-FlgE) are packed along the 11-start direction with the same head-to-tail manner (Figs. 6B and 6F), suggesting that the R-type hook likely functions as the hook of *S*. Typhimurium and represents the state of the hook under the thrust force generated by the filament during the CCW rotation of the polar flagellar motor and the forward swimming of the bacteria, while the L-type hook represents the state of the hook under the pulling force from the filament upon the rotation of the motor in the CW direction to pull the bacterial cell backward.

**Figure 6.**
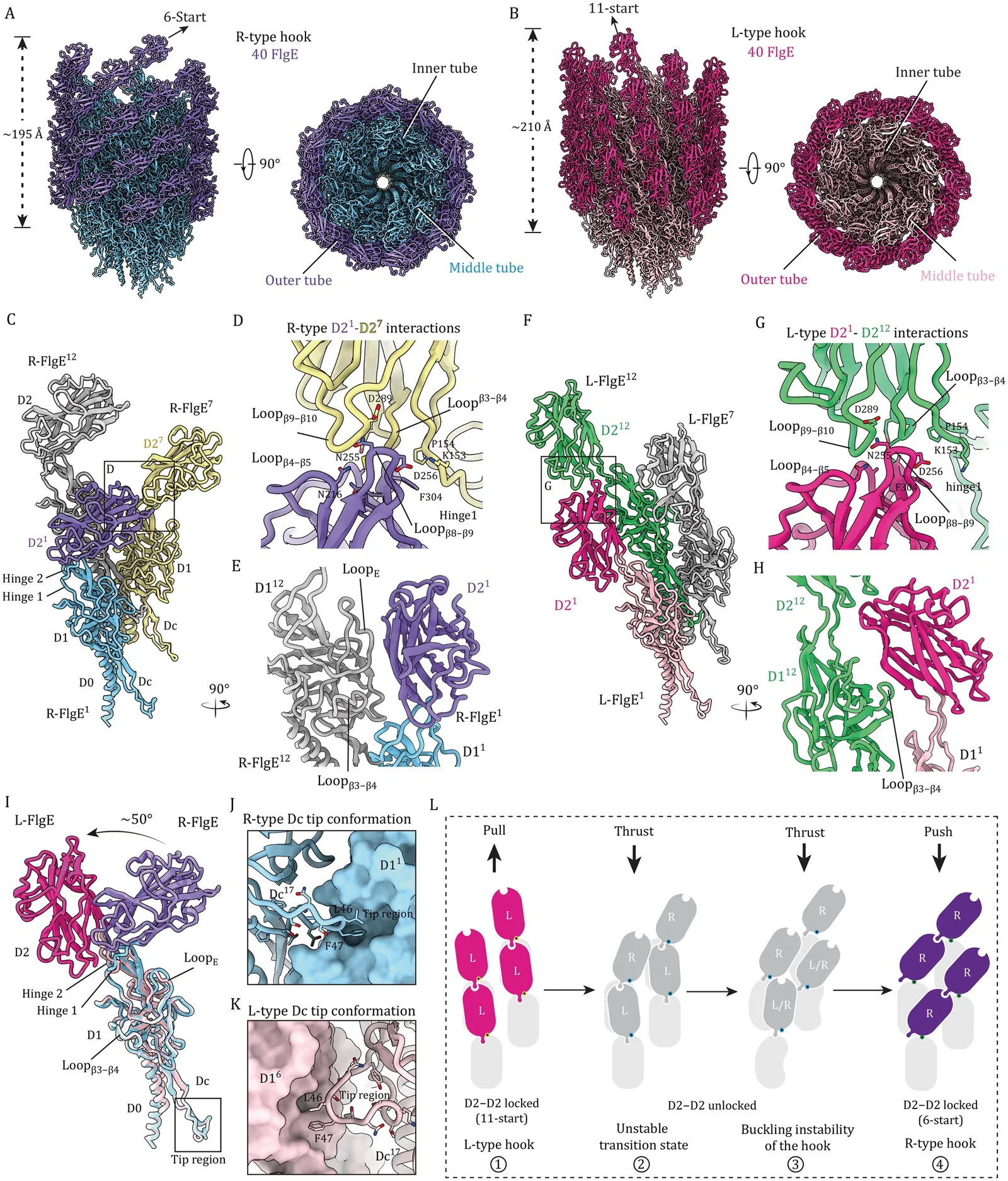
**Two types of hooks in the polar flagellar motor.** (A) Structure of the R-type hook. 40 FlgE subunits of the hook are illustrated in a cartoon. The outer tube of the hook is colored in deep purple. The middle and inner tubes of the hook are colored in cyan. (B) Structure of the L-type hook. The outer tube of the hook is colored in deep red. The middle and inner tubes of the hook are colored in light red. (C) Interactions of FlgE with adjacent subunits in the R-type hook. (D) Detailed interactions of the D2 domains of the FlgE subunits along the 6-start direction in the R-type hook. (E) Detailed interactions of the D2 domain with the D1 domains of the FlgE subunits along the 11-start direction in the R-type hook. (F) Interactions of FlgE with adjacent FlgE subunits in the L-type hook. (G) Detailed interactions of the D2 domains of the FlgE subunits along the 6-start direction in the L-type hook. (H) Detailed interactions of the D2 domain with the D1 domains of the FlgE subunits along the 11-start direction in the L-type hook. (I) Structural comparison of the FlgE subunit (L-FlgE) in the L-type hook with that (R-FlgE) in the R-type hook. The conformational change of the D2 domain is highlighted with a black arrow. The loop regions of the Dc domains (Dc-loop) are labeled with black lines. (J and K) Comparisons of the conformations and inter-subunit interfaces of the Dc-loops of R-FlgE (J) and L-FlgE (K). (L) Schematic diagram of a proposed mechanism for the bending and buckling instability of the hook in the “run-reverse-flick” motility pattern. Upon the rotation of the motor in the CW direction, the pulling force from the filament pulls the bacterial cell backward and generates the L-type hook (①). Once the rotational direction switching of the motor from CW to CCW, the thrust force from the filament likely disrupts D2–D2 interactions to allow the bending of the hook and avoid the structural collapses for the bacterial cell reorientation (② and ③). Following the steps, the D2 domains likely re-establish the D2-D2 interactions and assemble the R-type hook to push the forward swimming of the bacteria (④).

Different from *Salmonella* FlgE, *V. alginolyticus* FlgE has evolved a longer conserved tip region (L46-T52) in the Dc domain (Fig. S10A) and harbors unique loop_β3–β4_ and loop_E_ in the D1 domain, both of which are in the lifted conformations and located at the two sides of the D1 domain, respectively (Figs. 6E, 6H, and S10B). The longer Dc domains of the FlgE subunits of *V. alginolyticus* generate more extensive interactions between the Dc and D1 domains of adjacent subunits of FlgG or FlgE at the −5-, −11-, and −16-start positions in the middle tube (Figs. 6J, 6K, and S10C), thereby enhancing the rigidity of the hook in the polar motor and generating a tighter tubular junction between the rod and the hook. In addition, the hinge regions (hinge 1 and 2), which connect the D1 and D2 domains, in *V. alginolyticus* FlgE adopt conformations that are different from those in *Salmonella* FlgE (Fig. S11). The D2 domain of *V. alginolyticus* FlgE contains two long loops, loop_β5–β6_ and loop_β7–β8_ (Fig. S10D).

Structural comparison of the two types of the hook revealed that in the R-type hook, the head of the D2 domain of R-FlgE interacts with the loops at the tail of the D2 domain and the hinge1 and 2 regions of the next R-FlgE subunit along the 6-start direction (Figs. 6D and S10E). The loop_E_ of the D1 domain at the 11-start position binds to the upper surface of the D2 domain to stabilize the D2-domain protomers in the outer tube of the R-type hook (Fig. 6E). The loop_β3–β4_ regions have no interactions with the D2 domains (Fig. 6E). In the L-type hook, although the D2 domains are packed via the same surfaces that are used in the R-type hook (Fig. S10F), the D2 domains have no interactions with the hinge1 and 2 regions of the L-FlgE subunits along the 6-start direction (Fig. 6G). Instead of loop_E_, loop_β3–β4_ of the D1 domain at the 11-start position binds to the surface of the D2 domain to lock the conformation of the D2-domain protomers (Fig. 6H), suggesting that loop_E_ and loop_β3–β4_ function as anchors for the outer tube of the L- and R-type hooks, respectively. The tip region of the Dc domain of R-FlgE interacts with the D1 domain at the −16-start position (Figs. 6I and 6J), while the tip region of L-FlgE rotates to interact with the D1 domain at the −5-start position (Fig. 6K). The conformational changes of the tip region of the Dc domain indicate that switching from the L-type to R-type hook can induce the transient disruption of the tight D1–D1 and Dc–D1 interactions in the inner and middle tubes and provide structural flexibility to allow the bending of the hook. In addition, relative to that in L-FlgE, the D2 domain of R-FlgE undergoes a rotation by ∼50° via the hinge loops (Figs. 6I and S10G), which shortens the distances between the D2 domain protomers in the outer tube of the R-type hook and can avoid the structural collapse of the D2 domains in the innermost surface during the bending of the hook.

## Discussion

Bacterial flagellar motors have high diversity in structures for motility in various environments. This study reveals that the polar flagellar motor of *V. alginolyticus* has evolved many distinctive structural elements for high-torque transmission. The additional HT ring tightly binds to the LP ring, which exerts more electrostatic interactions and less physical contacts with the rod to reduce structural obstacles and energy consumption for its higher-speed rotation and high-torque transmission. Our findings are complemented by recent *in situ* cryo-EM studies of *C*. *jejuni* (Drobnic et al., 2025), which reported additional disk structures (FlgP- and FlgL-rings) around the LP and MS rings. Collectively, these results highlight the critical role of structural adaptations surrounding the LP ring in polar flagellar motors. Furthermore, the high-resolution structure of our study precisely defines a new conserved component FlrP the *Vibrio* polar flagellar motor and the two conformations (L- and R-type) of the hook, and uncovers the key roles of phospholipids in the rigidity maintenance of the rod. In contrast to the 11 rod-binding FliF peptides in the peritrichous flagellar motor (Tan et al., 2024), the MS ring of the polar flagellar motor extends 15 FliF peptide loops to bind the rod for torque transmission.

In the polar flagellar motor, there is also a new component, FlrP, which has a classic LRR structure with positive charges on the surface. The five FlrP subunits bind to the rod and the extended FliF peptide loops, and are located in the space between the P ring and the β-collar region of the MS ring. It was observed that the LPHT ring had slight sliding movements along the rod (Zhu et al., 2017). The protein size and positively-charged surface of FlrP are well complementary to the space and the lower negatively charged surface of the P ring, respectively, enabling the FlrP subunits to function as a spacer to prevent large movements of the LPHT ring and provide a cushioning action for the MS and the LPHT rings against the thrust or pulling force when the bacterial cell swims forward or undergoes the flicking process for the cell reorientation.

There are two types of hooks in the polar flagellum. The packing manners of the D2 domains of the FlgE subunits in the R-type hook can avoid the structural collapse of the D2 domains on the innermost surface during the bending of the hook. It is possible that the bacterial flicking process requires the state change of the hook from L-type to R-type (Fig. 6L). Upon the motor rotating in the CW direction, the pulling force from the filament pulls the bacterial cell backward and generates the L-type hook by the binding of the loop_β3–β4_ to the D2 domain and the D2–D2 interactions (Fig. 6L). Upon the switching of the rotational direction of the motor to CCW, the thrust force from the filament likely induces the disruption of the D2–D2 and D2–loop_β3–β4_ interactions in the L-hook and reduces the tip-mediated D1–D1 and Dc–D1 interactions to allow the bending of the hook for the bacterial cell reorientation. The D2 domains of FlgE subunits undergo a rotation by ∼50° via the hinge loops to avoid the structural collapses in the innermost side of the hook. After being bound by loop_E_ and hinge 1 and 2 of the D1 domains, the D2 domains likely re-establish the D2–D2 interactions and assemble the R-type hook to push the forward swimming of the bacteria (Fig. 6L). In contrast to the *Salmonella* FlgE, FlgE of *V. alginolyticus* has evolved many structural features, which render the hook not only a joint to connect the filament, but also a rudder for bacterial cell reorientation in swimming.

## Supplementary Material

pwag025_Supplementary_Data

## Data Availability

The cryo-EM density maps and structural models of the Vibrio polar flagellar motor-hook complexes have been deposited in the EMDB and PDB with the following accession numbers: the LPHT ring structure, EMDB: EMD-39776, PDB: 8Z5N; the Rod-Export apparatus partial hook complex, EMDB: EMD-39782, PDB: 8Z5U; the Proximal rod-Export apparatus, EMDB: EMD-39780, PDB: 8Z5S; the partial Rod, EMDB: EMD-39786, PDB: 8Z5W; the β-collar-RBM3 subrings of the MS ring with C34 symmetry, EMDB: EMD-39783, PDB: 8Z5V; the MS ring (C1, containing partial proximal rod and export apparatus), EMDB: EMD-39787, PDB: 8Z5X; the L-type hook, EMDB: EMD-39788, PDB: 8Z5Y; the R-type hook, EMDB: EMD-39789, PDB: 8Z5Z; and the overall polar flagellar motor-hook complex with C100 symmetry, EMDB: EMD-39790, PDB: 8Z60.

## Code availability

This paper does not report original code.
