# Supplementary material for: Molecular basis of high-torque transmission of the Vibrio polar flagellar motor
Source: Protein Cell. 2026 Mar 23;17(10):954–66. doi: 10.1093/procel/pwag025 (PMC13626733; doi:10.1093/procel/pwag025)
Supplement: Supplementary file 1 [file pwag025_supplementary_data.zip › P-C-10-2025-0689.R1-supplementary-final.docx]

**Materials and Methods**

**Bacterial strains and culture conditions**

The *Vibrio alginolyticus* strains VIO5 (Okunishi et al., 1996) and KK148 (Kusumoto et al., 2006; Kusumoto et al., 2008) were cultured at 30°C in VC medium consisting of 0.5% (w/v) tryptone, 0.5% (w/v) yeast extract, 3% (w/v) NaCl, 0.4% (w/v) K_2_HPO_4_, and 0.2% (w/v) glucose. *Escherichia coli* stains DH5α λπ and SM10 λπ were grown at 37°C in 2YT medium. Chloramphenicol and ampicillin were added at the final concentrations of 25 μg/mL and 10 μg/mL, respectively.

**Construction of *V. alginolyticus* mutant strains**

The *V. alginolyticus* KK148 strain is a multipolar flagellar mutant strain derived from the VIO5 strain with a *flhG* gene mutation, producing several polar flagella at one cell pole (Kusumoto et al., 2006). To eliminate the flagellar filament contamination during purification, the *flgL* gene, which encodes hook-associated protein 3 (HAP3) for connecting the filament to the hook, was deleted by using a homologous recombination-based gene knockout system (Green et al., 2019; Tan et al., 2021). The 1000 bp sequences upstream and downstream of the *flgL* coding region from the KK148 strain were amplified as the homologous arms to construct the pDM4-*ΔflgL* suicide plasmid. This plasmid includes a chloramphenicol resistance gene and an R6K replication origin dependent on the π protein. To avoid disrupting adjacent genes, 60 bp on each side of the *flgL* gene were retained. The obtained pDM4-*ΔflgL* plasmid was transformed into the *E. coli* SM10 λπ donor strain. The suicide plasmid-containing SM10 λπ strain and the recipient KK148 strain were separately cultured in antibiotic-free 2YT and VC medium, respectively, for six hours. The suspension cultures were mixed and deposited onto the 0.22 μm filter membrane placed on VC plate and incubated overnight at 30°C for conjugation. The mated culture was diluted and spread on VC plates containing 10 μg/mL chloramphenicol and 100 μg/mL ampicillin, then incubated overnight at 30°C for the first-round recombination. Single colonies were then grown in VC medium at 30°C and identified using primers specific to the *sacB* gene. The *sacB*-positive clones were streaked onto the sucrose plates containing 10% sucrose, 1% tryptone, 30 mM NaCl, 55 mM KCl and 1.25% agar, and incubated overnight at 30°C for the second-round recombination. The obtained mutant stain KK148-*ΔflgL* was finally validated by PCR and sequencing. The *V. alginolyticus* VIO5 strain is a lateral flagella-deficient strain with a single polar flagellum at the cell pole. The VIO5-*ΔflrP* strain was constructed using the same methods described for the construction of the KK148-*ΔflgL* stain. To construct the *V. alginolyticus* VIO5-Δ*flrP::flrP* mutant strain, the *flrP* gene was complemented into the genome of VIO5-*ΔflrP* strain using the pDM4 suicide vector.

**Purification of the polar motor-hook complex**

The KK148-*ΔflgL* strain was cultured overnight at 30°C in 5 mL of VC medium. The 1.6 mL of the culture was inoculated into 1 L of VC medium supplemented with 0.1% glucose and grown at 30°C for 4.5 h. The cells were harvested by centrifugation at 4,000 rpm and resuspended in 100 mL of lysis buffer consisting of 100 mM Tris-HCl (pH 8.0), 0.5 M sucrose, 10 mM EDTA (pH 7.5), and 0.1 mg/mL lysozyme. The suspension was stirred on ice at 200 rpm for 1 h. The cells were then lysed by adding Triton X-100 to a final concentration of 1% (v/v) and 20 mM MgSO_4_ at room temperature. To digest endogenous nucleic acids and clarify the cell lysates, 200 mg DNase I powder (BBI Life Sciences Corporation) was added to the mixture and incubated at room temperature for 10 minutes. The additional 100 mL of lysis buffer pre-mixed with 1% (v/v) Triton X-100 was then added, followed by incubation at room temperature for 10 minutes and at 4°C for 1 h. The cell debris was removed by centrifugation at 14,600 rpm for 20 min. The pH value of the supernatant was adjusted to 10.5 using 5 M NaOH, and denatured proteins were then removed by centrifugation at 14,600 rpm for 15 min. Subsequently, the supernatant was centrifugated at 35,000 rpm for 1 h at 4°C to obtain the membrane pellet containing the bacterial polar flagellar motor-hook complex particles. The pellet was resuspended in 3 mL of TET buffer (10 mM Tris-Cl, pH 8.0; 5 mM EDTA, pH 7.5; 0.1% Triton X-100) and further homogenized using a Dounce homogenizer (7 mL Tissue Grinder, Dounce, #357542). The suspension was incubated at 4°C for 1 h and clarified by centrifugation at 12,000 rpm for 10 min. The supernatant was then loaded onto a 20%-50% sucrose gradient in SW41 tubes and centrifuged at 20,000 rpm (~68,000 g) for 12 h at 4°C. The fractions containing the bacterial flagellar motor-hook complex particles were collected from the bottom of the tube in 200 μL aliquots. These fractions were analyzed by negative staining, and those containing the bacterial polar flagellar motor-hook complex were then pelleted by centrifugation at 20,000 rpm for 2 h. The transparent pellet was resuspended in 10 μL of TET buffer and immediately used for subsequent cryo-EM analyses for the motor-hook complex. The purification method of the LPHT ring was identical to that for the motor-hook complex, except that the double-distilled water, not the TET buffer, was used to prepare the sucrose gradient in centrifugation for isolating the disassociated LPHT ring particles.

**Negative staining analysis**

The 2.5 μL of the samples were loaded to a glow-discharged carbon-coated copper grid (200 mesh) for 2 min at room temperature, after which the solution was removed using filter paper. The grid was stained with 5 μL of 2% (w/v) uranyl acetate for three times, 10 s for the first and second staining, and 1 min for the third staining. The grids were air-dried at room temperature after removing the uranyl acetate. Grids were visualized using a transmission electron microscope (HT7700, HITACHI) operated at 80 kV.

**Cryo-EM sample preparation**

The 3 μL of the samples were applied onto the ultra-thin carbon film supported grids (QUANTIFOIL R1.2/1.3 300 Cu, 2nm) and then plunge-frozen using an FEI Vitrobot Mark IV. Before use, the grids were glow-discharged by PELCO easiGlow with the condition of 10 mA for 10 s. Blotting was performed for 3 s with a blotting force of 15, followed by a 60-second wait time and 2-second drain time at 100% humidity and 4°C. These parameters were applied for the sample preparation of the motor-hook complex and the LPHT ring. All these cryo-EM grids were then stored in liquid nitrogen for data collection.

**Cryo-EM data collection**

Data collection was conducted using FEI Titan Krios electron microscope operated at voltage of 300 kV equipped with a Falcon 4 camera. For the cryo-EM images of the motor-hook complex that mainly contain the end-on views of the disassociated LPHT ring particles, images were recorded using the software of EPU (Thermo Fisher Scientific) at a nominal magnification of 105,000. A total of 3,600 electron-event representation (EER) movies were acquired with defocus value ranging from 1.2 to 1.8 μm. The calibrated pixel size was set to 1.2 Å/pixel and the exposure time was set to 6.86 seconds, resulting in a total exposure dose of ~50 electrons/Å^2^. For the cryo-EM images of the LPHT ring that mainly contain the particles of the intact polar flagellar motor-hook complex, a total of 11,887 EER movies were captured using the same parameters.

**Cryo-EM Image processing**

The total of EER movies were imported into the CryoSPARC software (Punjani et al., 2017), followed by the motion correction and contrast transfer function (CTF) estimation. For the cryo-EM images of the motor-hook complex (3,684 micrographs), 3,284 particles of the LPHT ring in end-on views were obtained using template-based auto-picking with a box size of 560×560 pixels. Preliminary 2D averages revealed the 26-fold symmetry of the LPHT ring. For the cryo-EM images of the LPHT ring (11,887 micrographs), which contained the particles of the intact polar flagellar motor-hook complex, a total of 329,955 particles centered on the LPHT ring were automatically picked with a box size of 620×620 pixels. After multiple rounds of 2D classification, 61,395 particles were selected for ab-initio three-dimensional (3D) reconstruction. Subsequent homogeneous refinement applied with C26 symmetry produced a 2.8-Å density map of the LPHT ring. Due to the mixed conformations and the poor quality of the T ring at the bottom of the LPHT ring, we subtracted the signal of the central axial structure with the MS ring, and performed unsupervised 3D classification (without alignment, 4 classes) on the T ring region after non-uniform refinement (C26). Two main classes revealed that T ring has 13-fold symmetry, while the LPH ring has 26-fold symmetry. A total of 45,233 particles (73.8%) were subjected to the non-uniform refinement applied with C13 symmetry, yielding a 2.95-Å density map of the LPHT ring. We then removed the signal of the LPHT ring, which affected the overall structure calculation from 61,395 particles. Another round of 2D classification yielded 58,419 particles for ab-initio 3D reconstruction (C1) and non-uniform refinement (C1), generating a 3.5-Å density map of the region containing the hook, the rod, the export apparatus and the MS ring. Further CTF and local refinements produced a 3.0-Å density map of the region containing the export apparatus, the rod and a part of the hook (FlgE^1-11^-D0-D1 domains), and an additional local refinement produced a 3.0-Å density map of the proximal rod with the export apparatus. We then re-centered particles on the MS ring and produced a 3.6-Å density map of the MS ring region containing the MS ring, the export apparatus and a part of the proximal rod after homogeneous and local refinements with C1 symmetry. Further local refinement of the β-collar-RBM3 subrings with C34 symmetry improved its resolution to 2.9-Å.

A 3D classification (without alignment, 4 classes) focusing on the proximal rod region was performed due to the five extra densities observed near the proximal rod and the MS ring in the 3.5-Å density map of the region containing the hook, the rod, the export apparatus and the MS ring. Further CTF and local refinements of 19,450 particles (33.3%) in one class, featuring two β-solenoid-shaped densities, yielded a middle rod density map at 3.5-Å, with two β-solenoid structures bound to the outer surface of the proximal rod. Subsequently, all particles were re-centered on the hook region and re-extracted with a box size of 380×380 pixel. After 2D classification yielding 57,802 particles, two distinct conformations of the hook were identified: 34,667 particles of the L-type hook and 20,187 particles of the R-type hook. Ab initio reconstruction and nun-uniform refinement with C1 symmetry were performed, followed by 3D classification (without alignment, 4 classes) and helical refinements, generating a 3.2-Å density map of the L-type hook and a 3.4-Å density map of the R-type hook. Finally, a total of 61,395 particles were subjected to homogeneous refinement applied with C100 symmetry and produced an overall density map of the polar flagellar motor-hook complex, which served as the reference coordinate for the composite density maps and model building.

All map sharpening was performed automatically by CryoSPARC, which also generated the Fourier Shell Correlation (FSC) curves for the reconstructions. Average resolutions were estimated from the corrected FSC curves at the 0.143 threshold. Local resolution maps were calculated in CryoSPARC using the two independent half-maps from each reconstruction as input and visualized with *ChimeraX* (Goddard et al., 2018). All volume masks for local refinements were created using *Chimera* (Pettersen et al., 2004).

**Structure prediction by AlphaFold2**

Structure predictions of FlrP for model building were calculated using ColabFold (<https://github.com/sokrypton/ColabFold>) plugged in *ChimeraX*. A total of five models were generated for each prediction, and the best model was selected as an initial model for model building. Predicted structures were visualized and colored in *ChimeraX* based on the predicted local distance difference test (pLDDT) scores. The predicted aligned error (PAE) plot, which indicates the expected positional error of the predicted complex, was automatically generated by AlphaFold2 (Jumper et al., 2021).

**Model building and validation**

The structures of the FlgH, FlgI, FlgT, FlgPc, MotX and MotY monomers were manually modeled based on the initial model generated by *ModelAngelo* (Jamali et al., 2024) and refined in the 2.95-Å density map of the LPHT ring (C13), and produced the protomers of the LPH ring (FlgH_1_-FlgI_1_-FlgT_1_-FlgPc_1_) and the T ring (MotX_1_-MotY_1_, MotX_2_-MotY_2_). After multiple rounds of real-space refinement in *Phenix* (Adams et al., 2010) with manual inspection and adjustments in *Coot* (Emsley et al., 2010), the final models of the LPH ring and the T ring were generated in *ChimeraX* using C26 and C13 symmetry, respectively. The overall structure of the LPHT ring was obtained by combining the LPH ring and T ring after rigid-body refinement in *Phenix*. The structures of FliE, FlgF, FlgC, FlgB, FlgG, FliP, FliQ, FliR, FlhB, and FlgE-D0-D1 monomers were manually built and refined in the 3.0-Å density map of the region contain the rod, the export apparatus and a part of the hook and the map of the proximal rod with the export apparatus in *Coot* and *Phenix*, respectively, based on the initial models generated by *ModelAngelo*. The 6 FliE, 5 FlgB, 6 FlgC, 5 FlgF, 27 FlgG, 5 FliP, 4 FliQ, 1 FliR, 1 FlhB and 11 FlgE-D0-D1 subunits were generated according to the helical symmetry in *ChimeraX*. The models of the proximal rod with the export apparatus and of the distal rod with a part of the hook were obtained after several iterations of manual adjustments in *Coot* and real-space refinement in *Phenix* within the corresponding density maps. The final overall model of the rod-export apparatus-partial hook was obtained by combining the above two refined models and imported into *Phenix* for an additional round of rigid-body refinement.

The protomer of the β-collar-RBM3 subrings was built and refined in the 2.9-Å density map of the MS ring (C34), while the protomer of the RBM2 subring was built and refined in the 3.6-Å density map of the MS ring with the export apparatus and a part of the proximal rod (C1), based on the initial models generated by AlphaFold2. The overall model of the β-collar-RBM3 subrings was obtained using C34 symmetry in *ChimeraX* and refined with NCS (non-crystallographic symmetry) restraints in *Phenix*. The model of the RBM2 subring (C23) were adjusted individually in *Coot* and refined without NCS restraints in *Phenix*. The final model of the MS ring-export apparatus-proximal rod was obtained by combining the refined models of the β-collar-RBM3 subrings, the RBM2 subring, the export apparatus and the part of the proximal rod, and then imported into *Phenix* for a round of rigid-body refinement in the 3.6-Å density map of the MS ring (C1). The models of the FliF-L_A_^1-5^, FliF-L_B_^1-5^, and FliF-L_C_^1-5^ peptide loops were built in the 3.0-Å density map of the proximal rod with the export apparatus and the 3.5-Å density map of the middle region of the rod, based on the initial model generated by *ModelAngelo*. The FlrP^1^ and FlrP^5^ models were built in the 3.5-Å density map of the middle region of the rod based on the initial model generated from *ModelAngelo* and AlphaFold2. The final models were adjusted in *Coot* and then refined using *Phenix*.

The model of the R-FlgE subunit in the R-type hook was manually adjusted in *Coot* and further refined in *Phenix* in the 3.4-Å density map of the R-type hook, based on the initial model generated by AlphaFold2. The initial model of the L-FlgE subunit in the L-type hook was derived from the R-FlgE subunit and refined using *Phenix* in the 3.2-Å density map of the L-type hook. The models of the L-type hook and R-type hook were generated using the helical symmetry command in *ChimeraX*, incorporating 40 L-FlgE and 40 R-FlgE subunits in their corresponding density maps, and individually adjusted in *Coot* and refined using *Phenix*. The model of the L-hook-rod-export apparatus was obtained by aligning the L-hook with the rod-export apparatus-partial hook and FlrP in the 3.0-Å density map of the region containing the rod, the export apparatus and the part of the hook. The MS ring, the export apparatus, and the part of the proximal rod were also modeled in this density map to generate the L-hook-rod-export apparatus-MS ring model. This model with hat of the LPHT ring was then fitted into the density map of the overall polar flagellar motor-hook complex (C100) using *ChimeraX*. The final overall model of the polar flagellar motor-hook complex was composited by combining these two models and validated using *MolProbity* without further refinements. Four PG and five PE molecules were manually fitted into the 3.0- Å density map of the proximal rod–export apparatus based on the EM density using *Coot,* followed by manual adjustment and refinement. The phospholipid models were then incorporated into the overall model and further refined using *Phenix*.

All structural models were validated using *Molprobity* (Williams et al., 2018). The statistical data of data collection, processing, model refinement and validation are listed in Table S1. Representative structures and electron density are shown in Fig. S2B. Structural superpositions were performed using the in *ChimeraX*. The electrostatic distribution was visualized in *ChimeraX.* All Figures were generated using *ChimeraX.*

**Mass spectrometric analysis**

The SDS-PAGE gel containing the protein sample band was cut and digested by trypsin at 37°C overnight with prior reduction and alkylation in 50 mM ammonium bicarbonate. The extracted peptides were then subjected to NSI source followed by tandem mass spectrometry (MS/MS) in Q ExactiveTM Plus (Thermo) coupled online to the UPLC. The resulting MS/MS data were processed using Proteome Discoverer 1.3. Tandem mass spectra were searched against NCBI database (GenBank: AP022861.1, GenBank: AP022862.1). Mass error was set to 10 ppm for precursor ions and 0.02 Da for fragmental ions. Carbamidomethyl on cysteine were specified as fixed modification and oxidation on Met was specified as variable modification. Peptide confidence was set at high, and peptide ion score was set > 20.

**Bacterial motility assay**

To compare the motility of the VIO5 strain, the VIO5-*ΔflrP* strain and the complementary VIO5-*ΔflrP::flrP* strain*,* all strains were inoculated in 5 mL of antibiotic-free VC medium and cultured overnight. A 1 μL aliquot of the overnight cultures was spotted onto the VPG soft agar plate containing 0.25% (w/v) agar, 1% (w/v) tryptone, 0.4% (w/v) K_2_HPO_4_, 3% (w/v) NaCl, and 0.5% (w/v) glycerol. The volume of the remaining strains was adjusted, based on their OD_600_ values. The plates were incubated at 30°C for 6 h, and the diameters of the swimming circles were measured using ImageJ. This experiment was repeated 10 times, and the data were analyzed using GraphPad Prism 9.0 software.

**Western blot**

To determine the presence of the FlrP protein, the western blot was performed using the purified polar flagellar motor-hook complex particles. The samples were mixed with SDS loading buffer and boiled at 95 °C for 5 min, then applied to a 15% SDS-PAGE gel. After electrophoresis, the proteins were transferred onto PVDF membranes. The custom-made FlrP monoclonal antibody (rabbit source, HUABIO, #HA722715) was diluted in the TBST buffer (1:5000) and incubated for 1 h at room temperature. The basic ECL light-emitting substrate was used for chemiluminescence after a 1-hour incubation at room temperature with goat anti-rabbit IgG conjugated to horseradish peroxidase (1:10000). The PVDF membrane was then stained with Coomassie blue to visualize the bands.

**Quantification and statistical analysis**

In Figure S1 and S2, the tight and corrected FSC curves of each reconstruction were calculated using a tight mask alone and a tight mask with noise substitution correction based on two independent half-maps in CryoSPARC. These curves were illustrated using GraphPad Prism 9.0. The resolution estimations of the cryo-EM density maps were calculated in CryoSPARC using the two independent half-maps of each reconstruction as input, based on the corrected FSC curves at the FSC=0.143 criterion. The model resolution in Table S1 was evaluated using *phenix.mtriage* from the model-based noise free map and the experimental map at the FSC=0.5 criterion. Angle and distance measurements were performed in *ChimeraX*.

**References**

Adams, P.D., Afonine, P.V., Bunkoczi, G., Chen, V.B., Davis, I.W., Echols, N., Headd, J.J., Hung, L.W., Kapral, G.J., Grosse-Kunstleve, R.W.*, et al.* (2010). PHENIX: a comprehensive Python-based system for macromolecular structure solution. Acta Crystallogr D Biol Crystallogr 66, 213-221.

Barrio-Hernandez, I., Yeo, J., Janes, J., Mirdita, M., Gilchrist, C.L.M., Wein, T., Varadi, M., Velankar, S., Beltrao, P., and Steinegger, M. (2023). Clustering predicted structures at the scale of the known protein universe. Nature 622, 637-645.

Emsley, P., Lohkamp, B., Scott, W.G., and Cowtan, K. (2010). Features and development of Coot. Acta Crystallogr D Biol Crystallogr 66, 486-501.

Goddard, T.D., Huang, C.C., Meng, E.C., Pettersen, E.F., Couch, G.S., Morris, J.H., and Ferrin, T.E. (2018). UCSF ChimeraX: Meeting modern challenges in visualization and analysis. Protein Sci 27, 14-25.

Green, C.A., Kamble, N.S., Court, E.K., Bryant, O.J., Hicks, M.G., Lennon, C., Fraser, G.M., Wright, P.C., and Stafford, G.P. (2019). Engineering the flagellar type III secretion system: improving capacity for secretion of recombinant protein. Microb Cell Fact 18, 10.

Jamali, K., Kall, L., Zhang, R., Brown, A., Kimanius, D., and Scheres, S.H.W. (2024). Automated model building and protein identification in cryo-EM maps. Nature 628, 450-457.

Jumper, J., Evans, R., Pritzel, A., Green, T., Figurnov, M., Ronneberger, O., Tunyasuvunakool, K., Bates, R., Zidek, A., Potapenko, A.*, et al.* (2021). Highly accurate protein structure prediction with AlphaFold. Nature 596, 583-589.

Okunishi, I., Kawagishi, I., and Homma, M. (1996). Cloning and characterization of motY, a gene coding for a component of the sodium-driven flagellar motor in Vibrio alginolyticus. J Bacteriol 178, 2409-2415.

Pettersen, E.F., Goddard, T.D., Huang, C.C., Couch, G.S., Greenblatt, D.M., Meng, E.C., and Ferrin, T.E. (2004). UCSF Chimera--a visualization system for exploratory research and analysis. J Comput Chem 25, 1605-1612.

Punjani, A., Rubinstein, J.L., Fleet, D.J., and Brubaker, M.A. (2017). cryoSPARC: algorithms for rapid unsupervised cryo-EM structure determination. Nat Methods 14, 290-296.

Williams, C.J., Headd, J.J., Moriarty, N.W., Prisant, M.G., Videau, L.L., Deis, L.N., Verma, V., Keedy, D.A., Hintze, B.J., Chen, V.B.*, et al.* (2018). MolProbity: More and better reference data for improved all-atom structure validation. Protein Sci 27, 293-315.

**Supplementary figures**

**Figure S1. Cryo-EM data processing of the polar flagellar motor-hook complex.**

(A) A representative cryo-EM micrograph of the polar flagellar motor-hook complex.

(B) A representative cryo-EM micrograph of the particles of the LPHT ring collected. Scale bar, 50 nm (A, B). (C) Fow chart of the cryo-EM data processing and structural determination of the polar flagellar motor-hook complex. (D) Flow chart of the cryo-EM data processing of the LPHT ring.

**Figure S2. FSC curves and representative cryo-EM density maps of the polar flagellar motor-hook complex.**

(A) The FSC (Fourier Shell Correlation) curves and estimated resolutions for the reconstructions in (Fig. S1). (B) Representative cryo-EM density maps of FlgH, FlgT, FlgI, MotY, FlgF, MotX, FliE, FlrP and FliF after local refinements.

**Figure S3. Structures of FlgH and FlgI in the polar LP ring.**

(A) Structural comparison of FlgH in the polar flagellar motor-hook complex with that in the peritrichous flagellar motor-hook complex. The polar and peritrichous FlgHs are colored in cyan and blue, respectively. (B) Inter-subunit interactions of FlgH in the L ring in the polar flagellar motor. The S1, S2 β-sheets and L_N_ loop of the polar FlgH are colored in pink, orange and cyan, respectively. (C) Close-up view of the L_N_ loop and its interactions with adjacent FlgH subunits in the L ring. (D) Sequence alignment of the L_N_ loop of FlgH of *V.* *alginolyticus* with those of other bacterial species. (E) Inter-subunit interactions of FlgI in the P ring. Each FlgI subunit interacts with the prior and next two FlgI subunits. (F) Structural comparison of FlgI in the polar flagellar motor-hook complex and that in the peritrichous flagellar motor-hook complex. The D1, RBM1 and RBM2 domains of the polar FlgI are colored in orange, yellow and green, respectively. The peritrichous FlgI is colored in cyan. (G) The 1-versus-3 interaction manner of the FlgH and FlgI subunits with each other between the L and P rings in the polar flagellar motor. (H) The 1-versus-4 interaction manner of the FlgH and FlgI subunits with each other between the L and P rings in the peritrichous flagellar motor.

(I) Bottom view of the structure of the LPH ring. (J) Sequence alignment of FlgI proteins from *V. alginolyticus* and *S.* Typhimurium. Loop2 and Loop3 of *Vibrio* FlgI are indicated. Four lysine residues of the *Vibrio* FlgI are highlighted with blue asterisks, whereas the interacting polar residues of *Salmonella* FlgI are highlighted with green asterisks.

**Figure S4. The symmetry mismatch in the LPHT ring structure.**

(A) Overall view of the structure of the T ring. Each protomer of the T ring consists of MotX_1_, MotY_1_, MotX_2_ and MotY_2_, which are colored in orange, cyan, gold and deep blue, respectively. The LPH ring is colored in grey. The subunits of FlgH, FlgI and FlgT in a protomer of the LPH ring are colored in cyan, yellow and red, respectively. (B) Close-up view of the protomer of the T ring. Each protomer of the T ring contains two MotX-Y heterodimers. (C) Structural comparison of the two MotY subunits of the protomer. The N-terminal (MotY-N) and C-terminal (MotY-C) domains of each subunit are indicated. (D) Structural comparison of the two MotX subunits of a protomer. The two C-terminal regions of MotX exhibit distinct conformations and are critical for the interactions of the two MotX-Y dimers in a protomer. (E) Interactions between MotX_1_ and MotY_1_ in the protomer. The CTD of MotY_2_ also interact with MotX_1_ - MotY_1_ protomer. (F-G) Detailed interactions of the CTD region of MotX_2_ with MotX_1_ and MotY_1_-C in a protomer via hydrophobic interaction. (H) Interaction of the N-terminal domains of MotY_1_ and MotY_2_ in a protomer. (I) Interactions between the MotY-N domain and the upper LPH ring. MotY-N inserts into the cleft between the FlgI-RBM1 and FlgT-C domains to stabilize the P ring. (J) Interactions of MotX_2_ with the MotY-N domains in a protomer. MotX interacts with the upper two MotY-N domains to bridge the two subrings of the T ring.

**Figure S5. Inter-subunit interactions in the H and T rings.**

(A) Structural comparison of FlgT in the polar flagellar motor-hook complex with its apo structure (PDB ID: 3W1E). FlgT consists of three domains (FlgT-N, FlgT-M, and FlgT-C) (*35*) and adopts a closed conformation, contrasting with the open conformation observed in the apo crystal structure. (B) Inter-subunit interactions of the FlgT subunits in the H ring. (C) Interactions of the N-terminal region of the L_N_ loop of FlgH with the H ring. The invisible lipid-modified N-terminal domain of the L_N_ loop likely anchors the LPHT ring to the outer membrane via the FlgP subring. (D) Structural superimposition of the MotX_1_-MotY_1_ and MotX_2_-MotY_2_ dimers of a protomer, based on the C-terminal domains of MotY. (E) The inter-protomer interaction of the C-terminus of MotY_2_ with the MotY_1_ subunit of the next protomer in the T ring.

**Figure S6. The export apparatus, and the structures of the rod components in the polar flagellar motor.**

(A) Structural comparison of the two N-terminal transmembrane helices of FlhB and FliQ. (B) Structural comparison of the proximal rod-binding export apparatus in the polar flagellar motor-hook complex with the free export apparatus (PDB ID: 6S3L). The free export apparatus is colored in grey. The export apparatus adopts an expanded configuration to accommodate the subunits of the proximal rod. (C) The three-layered tubular structure of the rod in the polar flagellar motor-hook complex. The subunits of FliE, FlgB, FlgC, FlgF and FlgG are colored in salmon, yellow, orange, purple and light green, respectively. The GSS region of FlgG proteins is highlighted in pink. (D-G) Structural comparison of the rod components in the polar and *Salmonella* peritrichous flagellar motor-hook complexes. (H) Interactions of the FlgG^11-27^-GSS region with adjacent subunits in the rod. (I-J) Comparison of the electrostatic potential surfaces of the LP ring (left) and the rod (right) in the polar and peritrichous flagellar motor-hook complexes. The L ring rod-facing surface and distal rod surface of the polar flagellar motor carry a higher abundance of negatively charged residues compared with those of the peritrichous flagellar motor. (K) Comparison of the numbers of the aspartic acid (Asp, D) and glutamic acid (Glu, E) residues on the surface of FlgH and the D1 domain of FlgG at the rod-LP ring interface. (L) Distribution of the aspartic acid and glutamic acid residues on the surfaces of the D1 domains of FlgGs of *V.* *alginolyticus* (left) and *S.* Typhimurium (right). (M) Two-view representation of the hydrophobic binding pocket for the first and second phospholipid molecules.

**Figure S7. Structure of the MS ring in the polar flagellar motor-hook complex.**

(A-B) Top (A) and bottom (B) views of cryo-EM density map of the MS ring constructed by C1 symmetry. The β-collar-RBM3 subrings have the C34 symmetry (A), while the RBM2 subring exhibits the C23 symmetry (B). (C) Interaction of the RBM2 domain of FliF with the export apparatus through Loop 1 and 2 in the polar flagellar motor-hook complex. (D) Top view of the distribution of the 15 FliF peptide loops from *V. alginolyticus*. The loops are grouped into 5 sets, each of which contains a L_A_, L_B_ and L_C_ loops. (E) Top view of the distribution of the 11 FliF peptide loops (PDB ID: 8WLH) from *S.* Typhimurium. The 11 FliF loops include 5 sets of L1 and L2 loops and one L3 loop.

**Figure S8. Identification of FlrP in the polar flagellar motor.**

(A) Mass spectrometry analysis of the components in the purified particles of the polar flagellar motor-hook complex. The leucine-rich repeat (LRR) protein FlrP is highlighted in red in the table. (B) Western blot examination of FlrP in the purified particles of the polar flagellar motor-hook complex. Upper, western blot; blow, SDS-PAGE with Coomassie blue staining. (C) Representative cryo-EM density map of FlrP with the rod. The densities of FlrP^1^ and FlrP^5^ are illustrated and colored in red. (D) The sequence consensus of the leucine-rich repeated coils of FlrP. (E) Cryo-EM density map of the five FlrP subunits bound to the rod. (F-G) Side and bottom views of the positions of the FlrP subunits under the P ring. (H) Cross-section view of the electrostatic potential distributions of the surfaces of the FlrP subunits and the P ring. (I) The top view of the electrostatic potential distributions of the five FlrP subunits. (J) The positively charged residues on the surface of FlrP. The surface is colored by relative electrostatic potential with red indicating negatively charged and blue positively charged (H-J).

**Figure S9. The effect of FlrP in the motility of *V. alginolyticus* and *V. parahaemolyticus*.**

(A) The effect of gene deletion of *FlrP* on the motility of *V. alginolyticus*. The swimming motility assay of the *V. alginolyticus* VIO5 strain was analyzed using VPG soft-agar plates containing 0.25% agar, quantified after a 6 h incubation at 30℃. Statistical analyses of the effect of gene deletion of *FlrP* on the motility of *V. alginolyticus* are listed at right. (B) The effect of gene deletion of *FlrP* on the motility of *V. parahaemolyticus*. Statistical analyses of the effect are listed at right. The swimming diameters of each sample were measured using ImageJ and normalized to the wild type (WT). Bar graphs represent the mean of at least ten biologically independent replicates with individual data points shown. Statistical significances were determined using one-way ANOVA followed by Dunnett’s post hoc test (****p < 0.0001) (A-B). (C) AFDB-based structural clustering of FlrP homologues in *Vibrionaceae* using Foldseek web server (Barrio-Hernandez et al., 2023). (D) Structural comparison of the FlrP homologues from *V. parahaemolyticus*, *V. cholerae* and *V. vulnificus.* (E-F) The ten replicate raw motility assay images used for panels (A) and (B) are shown for *V. alginolyticus* (F) and *V. parahaemolyticus* (F), respectively.

**Figure S10. Structural comparison of the FlgE subunits in the R- and L-type hooks.**

(A) Structural comparison of FlgE of *V. alginolyticus* in the R-type hook (R-*Va*FlgE) of the polar flagellar motor with the *Salmonella* FlgE (*Sa*FlgE) (*14*) of the peritrichous flagellar motor. The tip region is highlighted with dashed circle lines. (B) Structural comparison of the D1 domains of FlgE of *V. alginolyticus* and *Salmonella* Typhimurium. The loop_β3-β4_ and loop_E_ of the D1 domain of the polar FlgE are highlighted in red. (C) The interactions of the Dc loop of the FlgE subunit with the adjacent FlgE and FlgG subunits at the interface of the hook and the rod. (D) Structural comparison of the D2 domains of FlgE of *V. alginolyticus* and *Salmonella* Typhimurium. The distinctive loops in the D2 domains of FlgE of *V. alginolyticus* are labeled as indicated. (E) Detailed interactions of the D2 domains of the R-FlgE subunits along the 6-start direction in the R-type hook. (F) Detailed interactions of the D2 domains of the L-FlgE subunits along the 11-start direction in the L-type hook. (G) Conformational changes of the FlgE subunits in the L- and R-type hooks. The FlgE subunits in the L- and R-type hooks are labeled as L-FlgE and R-FlgE, respectively. The structures of FlgE are shown as ribbons. Compared with that of L-FlgE, the D2 domain of R-FlgE undergoes a ~50° rotation through the hinge loops.

**Figure S11. Sequences alignment of the FlgE proteins of *V. alginolyticus*** **and** ***S.* Typhimurium.** The unique regions in the polar FlgE, including hinge 1 and 2, loop_β3-β4_ and loop_E_ are highlighted. The D0, Dc, D1 and D2 domains are labeled as indicated.

**Table S1. Cryo-EM data collection, refinement and validation statistics.**

|  | **LPHT ring**  (EMDB-39776)  (PDB 8Z5N) | **Proximal rod- phospholipid-**  **export apparatus**  (EMDB-39780)  (PDB 8Z5S) | **Rod-export apparatus with**  **Partial hook**  (EMDB-39782)  (PDB 8Z5U) |
| --- | --- | --- | --- |
| **Data collection and processing** |  |  |  |
| Magnification | 105,000 | 105,000 | 105,000 |
| Voltage (kV) | 300 | 300 | 300 |
| Electron exposure (e–/Å^2^) | 50 | 50 | 50 |
| Defocus range (μm) | 1.2 to 1.8 | 1.2 to 1.8 | 1.2 to 1.8 |
| Pixel size (Å) | 1.2 | 1.2 | 1.2 |
| Symmetry imposed | C13 | C1 | C1 |
| Initial particle images (no.) | 329,955 | 329,955 | 329,955 |
| Final particle images (no.) | 45,233 | 58,418 | 58,418 |
| Map resolution (Å)  FSC threshold | 2.95  0.143 | 3.03  0.143 | 3.04  0.143 |
|  |  |  |  |
| **Refinement** |  |  |  |
| Initial model used (PDB code) | ModelAngelo | ModelAngelo | ModelAngelo |
| Model resolution (Å)  FSC threshold | 3.1  0.5 | 3.3  0.5 | 3.2  0.5 |
| Map sharpening *B* factor (Å^2^) | -96.7 | -58.1 | -58.7 |
| Model composition  Non-hydrogen atoms  Protein residues  Ligands | 269,711  34,671  N/A | 38,700  5,016  9 | 118,072  15,597  N/A |
| *B* factors (Å^2^)  Protein  Ligand | 50.43  N/A | 52.23  56.76 | 52.32  N/A |
| R.m.s. deviations  Bond lengths (Å)  Bond angles (°) | 0.004  0.528 | 0.003  0.559 | 0.003  0.544 |
| Validation  MolProbity score  Clashscore | 1.83  8.79 | 1.87  8.07 | 1.93  7.86 |
| Ramachandran plot  Favored (%)  Allowed (%)  Disallowed (%) | 97.90  1.99  0.11 | 97.94  2.00  0.06 | 97.56  2.32  0.12 |

|  | **β-collar-RBM3**  **subrings of the**  **MS ring (C34)**  (EMDB-39783)  (PDB 8Z5V) | **MS ring with the proximal rod and the export apparatus (C1)**  (EMDB-39787)  (PDB 8Z5X) | **L-hook**  (EMDB-39788)  (PDB 8Z5Y) |
| --- | --- | --- | --- |
| **Data collection and processing** |  |  |  |
| Magnification | 105,000 | 105,000 | 105,000 |
| Voltage (kV) | 300 | 300 | 300 |
| Electron exposure (e–/Å^2^) | 50 | 50 | 50 |
| Defocus range (μm) | 1.2 to 1.8 | 1.2 to 1.8 | 1.2 to 1.8 |
| Pixel size (Å) | 1.2 | 1.2 | 1.2 |
| Symmetry imposed | C34 | C1 | Helical |
| Initial particle images (no.) | 329,955 | 329,955 | 329,955 |
| Final particle images (no.) | 58,418 | 58,418 | 32,950 |
| Map resolution (Å)  FSC threshold | 2.95  0.143 | 3.64  0.143 | 3.22  0.143 |
|  |  |  |  |
| **Refinement** |  |  |  |
| Initial model used (PDB code) | ModelAngelo | ModelAngelo  AlphaFold2 | AlphaFold2 |
| Model resolution (Å)  FSC threshold | 3.2  0.5 | 6.2  0.5 | 3.5  0.5 |
| Map sharpening *B* factor (Å^2^) | -109.9 | -52.1 | -77.7 |
| Model composition  Non-hydrogen atoms  Protein residues  Ligands | 39,814  5,032  N/A | 88,328  11,398  N/A | 132,760  17,400  N/A |
| *B* factors (Å^2^)  Protein  Ligand | 44.26  N/A | 58.09  N/A | 65.49  N/A |
| R.m.s. deviations  Bond lengths (Å)  Bond angles (°) | 0.003  0.479 | 0.004  0.563 | 0.005  0.613 |
| Validation  MolProbity score  Clashscore | 1.88  5.21 | 2.31  9.75 | 2.05  6.22 |
| Ramachandran plot  Favored (%)  Allowed (%)  Disallowed (%) | 97.18  2.82  0.00 | 96.05  3.71  0.24 | 96.65  3.22  0.13 |

|  | **R-hook**  (EMDB-39789)  (PDB 8Z5Z) | **FlrP-rod-FliF**  **Peptide loops**  (EMDB-39786)  (PDB 8Z5W) | **The polar flagellar motor-hook complex**  (EMDB-39790)  (PDB 8Z60) |
| --- | --- | --- | --- |
| **Data collection and processing** |  |  |  |
| Magnification | 105,000 | 105,000 | 105,000 |
| Voltage (kV) | 300 | 300 | 300 |
| Electron exposure (e–/Å^2^) | 50 | 50 | 50 |
| Defocus range (μm) | 1.2 to 1.8 | 1.2 to 1.8 | 1.2 to 1.8 |
| Pixel size (Å) | 1.2 | 1.2 | 1.2 |
| Symmetry imposed | Helical | C1 | C100 |
| Initial particle images (no.) | 329,955 | 329,955 | 329,955 |
| Final particle images (no.) | 19,402 | 19,450 | 61,395 |
| Map resolution (Å)  FSC threshold | 3.42  0.143 | 3.46  0.143 | 3.22  0.143 |
|  |  |  |  |
| **Refinement** |  |  |  |
| Initial model used (PDB code) | AlphaFold2 | ModelAngelo |  |
| Model resolution (Å)  FSC threshold | 3.9  0.5 | 3.7  0.5 |  |
| Map sharpening *B* factor (Å^2^) | -61.2 | -44.8 |  |
| Model composition  Non-hydrogen atoms  Protein residues  Ligands | 132,760  17,400  N/A | 36,128  4,810  N/A |  |
| *B* factors (Å^2^)  Protein  Ligand | 65.49  N/A | 54.19  N/A |  |
| R.m.s. deviations  Bond lengths (Å)  Bond angles (°) | 0.002  0.416 | 0.003  0.525 |  |
| Validation  MolProbity score  Clashscore | 1.75  5.84 | 1.92  7.64 |  |
| Ramachandran plot  Favored (%)  Allowed (%)  Disallowed (%) | 97.74  2.06  0.00 | 97.24  2.63  0.13 |  |
